# Phagosome Escape of Rough *Mycobacterium abscessus* Strains in Murine Macrophage via Phagosomal Rupture Can Lead to Type I Interferon Production and Their Cell-To-Cell Spread

**DOI:** 10.3389/fimmu.2019.00125

**Published:** 2019-01-31

**Authors:** Bo-Ram Kim, Byoung-Jun Kim, Yoon-Hoh Kook, Bum-Joon Kim

**Affiliations:** Department of Microbiology and Immunology, Biomedical Sciences, Liver Research Institute and Cancer Research Institute, College of Medicine, Seoul National University, Seoul, South Korea

**Keywords:** *Mycobacterium abscessus*, phagosomal escape, rough strains, type I interferon, phagosomal rupture, cell death, cell-to-cell spread

## Abstract

*Mycobacterium abscessus* complex (MAB) is a rapidly growing mycobacterium(RGM) whose clinical significance as an emerging human pathogen has been increasing worldwide. It has two types of colony morphology, a smooth (S) type, producing high glycopeptidolipid (GPL) content, and a rough (R) type, which produces low levels of GPLs and is associated with increased virulence. However, the mechanism responsible for their difference in virulence is poorly known. By ultrastructural examination of murine macrophages infected, we found that MAB-R strains could replicate more actively in the macrophage phagosome than the S variants and that they could escape into cytosol via phagosomal rupture. The cytosolic access of MAB-R strains via phagosomal rupture led to enhanced Type I interferon (IFN) production and cell death, which resulted in their cell-to-cell spreading. This behavior can provide an additional niche for the survival of MAB-R strains. In addition, we found that their enhancement of cell death mediated cell spreading are dependent on Type I IFN signaling via comparison of wild-type and IFNAR1 knockout mice. In conclusion, our data indicated that a transition of MAB-S strains into MAB-R variants increased their virulence via enhanced Type I IFN production, which led to enhanced survival in infected macrophage via cell death mediated cell-to-cell spreading. This result provides not only a novel insight into the difference in virulence between MAB-R and -S variants but also hints to their treatment strategy.

## Introduction

*Mycobacterium abscessus* complex (MAB) is now recognized as a major pathogen leading to pulmonary infection within the rapidly growing mycobacteria (RGMs) (1–3) and is a common pathogen in lung diseases, especially in cystic fibrosis patients (4–6). In South Korea, MAB lung diseases have also been increasing in frequency and account for 70~80% of RGM-induced lung diseases (7, 8). MAB is also one of the major pathogens leading to nosocomial infections (9). MAB infections are difficult to treat due to both natural broad-spectrum resistance and acquired resistance, with disparate antibiotic susceptibility patterns being observed between different clinical strains (10, 11).

MAB consists of diverse subspecies or genotypes. Currently, the MAB group can be divided into two subspecies, *M. abscessus* subsp. *abscessus* (hereafter, S-Abs) and *M. abscessus* subsp. *bolletii. M. abscessus* subsp. *bolletii* was proposed to include the two former species *M. massiliense* (S-Mas) and *M. bolletii* (S-Bol) (12, 13). S-Mas can be further subdivided into two *hsp*65 genotypes (Type I and Type II) (14).

MAB consists of two phenotypes: smooth colony (MAB-S) and rough colony (MAB-R), with or without glycopeptidolipid (GPL) gene synthesis (15, 16). In relation to these phenotypes, there have been reports of infection experiments using various cells such as macrophages and bronchial epithelial cells, and studies on the etiology of the pathogenesis in mouse models (16–18). MAB-S has an advantage in survival due to GPL-based biofilm formation, leading to inhibit bacteria-induced apoptosis (2). So, the MAB-R variant without outer GPL induces could induce enhanced apoptotic cell death mediated invasion ability than MAB-S (17, 18). However, the underlying mechanism regarding the disparity between the pathogenic potential of MAB-R and -S types remains unknown.

Virulent *Mycobacterium tuberculosis* (*Mtb*) strains have ability to escape into the cytosol from the phagosome via the type VII secretion system ESX-1 (19, 20), which is responsible for the secretion of the 6-kDa early secreted antigenic target (ESAT-6), and its protein partner, the 10-kDa culture filtrate protein (CFP-10). *Mycobacterium marinum* can escape into the cytosol via a similar strategy as virulent *M. tuberculosis* (21). Active phagosomal rupture in antigen-presenting cells (APCs) such as macrophages or dendritic cells induced by the ESX-1 system present in the genome of pathogenic mycobacteria can expose bacterial DNA in the cytosol, which in turn drives the transcription of IFN-β via the cGAS–STING–TBK1–IRF3 axis and enhanced IL-1β secretion via NLRP3 inflammasome activation (3, 22). The activation of both Type I IFN signaling and inflammasome systems might synergistically contribute to the enhanced virulence of pathogenic mycobacteria via damping excessive inflammation and tissue damage. Furthermore, ESX-1–derived phagosomal rupture can result in toxicity and enhanced host cell death, also contributing to the virulence of pathogenic mycobacteria via increased intracellular bacterial growth(23–25).

Several previous studies consistently demonstrated that the MAB-R type survived more efficiently during infection into macrophage or dendritic cells than the MAB-S type (15, 18, 26, 27). Therefore, we hypothesized that enhanced survival of MAB-R strains in APCs may be due to the bacteria cytosol access and subsequent phagosomal rupture. However, the previous complete genome studies of several MAB strains revealed that no orthologs corresponding to *M. tuberculosis* ESX-1 genes are in their genomes (28), suggesting there may be an alternative strategy facilitating cytosol access of the MAB-R type. Here, we elucidated the underlying mechanism that likely explains the distinct pathogenic potentials between the MAB-R and -S types, mainly focusing on Type I IFN signaling of MAB-R strains, the MAB-R access to cytosol rupture and their enhanced survival in macrophage via host-cell death mediated cell-to-cell spreading.

## Results

### MAB-R Strains Showed Greater Intracellular Growth and Innate Immune Response in Murine Macrophage Than MAB-S Strains

**Figure 1:**
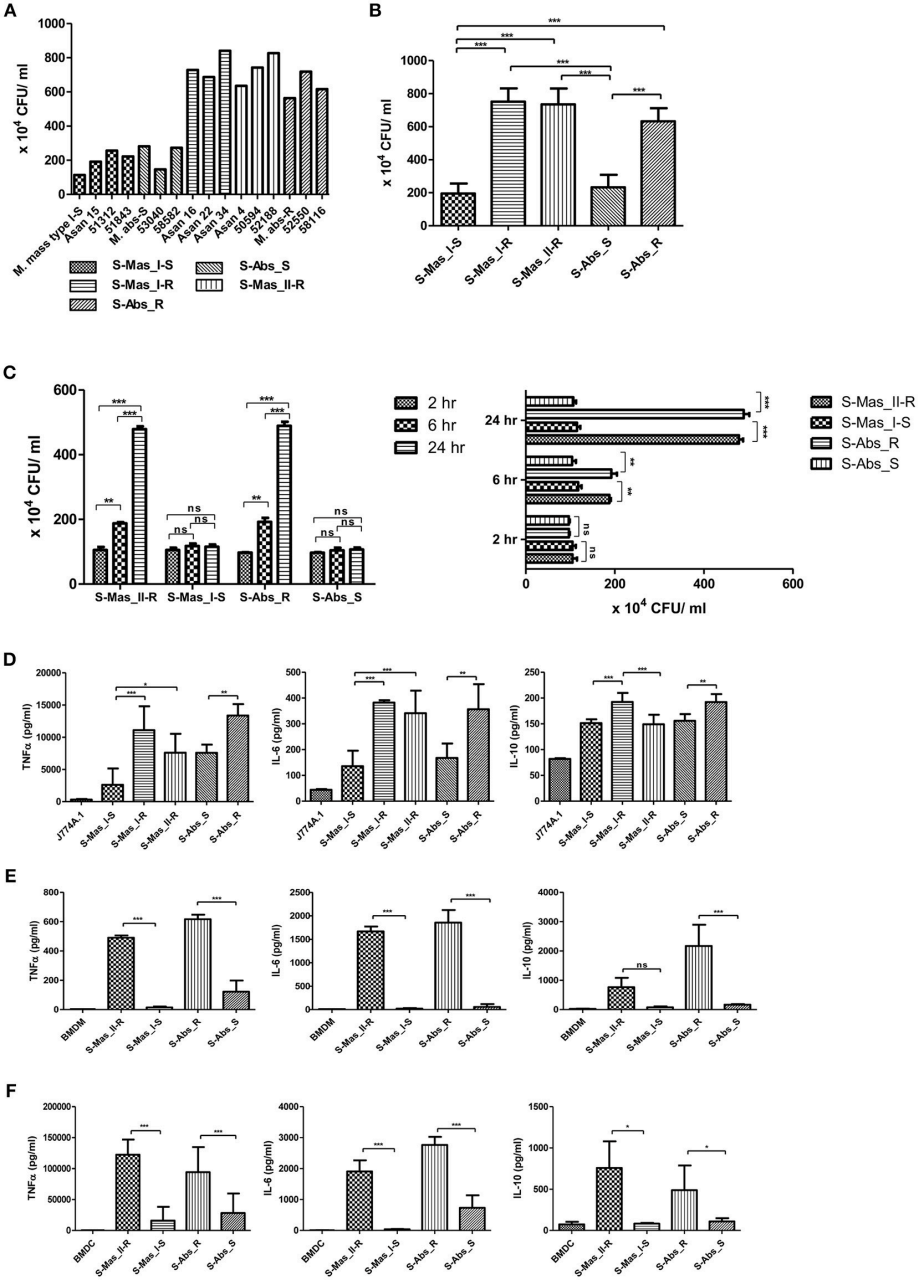
Comparison of intracellular survival and proinflammatory cytokine production of MAB-R and -S strains of various subspecies or genotypes in infected murine macrophage. **(A)** CFU assays of MAB-R and -S strains of various subspecies or genotype [S-Abs smooth strains (S-Abs_S): *M. abscessus* type strain ATCC 19977 smooth strain, Asan 53040, and Asan 58582; S-Abs rough strains (S-Abs_R): *M. abscessus* type strain ATCC 19977 rough strain, Asan 52550 and Asan 58116; S-Mas type I-Smooth (S-Mas_I-S): type strain, Asan 15, Asan 51312, and Asan 51843; S-Mas type I-Rough (S-Mas_I-R): Asan 16, Asan 22, and Asan 34; and S-Mas type II-Rough (S-Mas_II-R): Asan 4, Asan 50594, and Asan 62188] (1 × 10^7^ bacteria; 10 M.O.I.) in infected J774A.1 (1 × 10^6^) cells at 37°C for 24 h.p.i. **(B)** The mean number of CFUs of various subspecies or genotypes of MAB surviving in infected J774A.1 cells. **(C)** Comparison of the number of CFUs from MAB-R [S-Abs_R (*M. abscessus* type strain ATCC 19977 rough strain) and S-Mas_II-R (Asan 50594)] and MAB-S strains [S-Abs_S (*M. abscessus* type strain ATCC 19977 smooth strain) and S-Mas_I-S (Asan 51843)] at 37°C at different time points (2, 6, and 24 h) after infection of BMDM at 10 M.O.I. **(D–F)** Comparison of the mean value of proinflammatory cytokine production (TNF-α, IL-6, and IL-10) between various MAB-R and -S strains (10 M.O.I, at 24 h.p.i) from infected J774A.1 cells **(D)**, BMDMs **(E)**, and BMDCs **(F)**. The results are representative of two independent experiments and represent means ± *SD. P*-values were determined by one-way ANOVA with Tukey's multiple comparison test **(B,D–F)** and paired Student's *t*-test **(C)** using GraphPad prism program: ns, non-significant; *P < 0.05; **P < 0.01, and ***P < 0.001.

Previously, MAB-R strains have been reported to better survive in macrophage and lead to more proinflammatory cytokines than MAB-S strains (26). However, variation in survival- or inflammation-inducing capacity between subspecies or genotypes of MAB has not been addressed. Therefore, we evaluated the intracellular growth (Figures 1A–C) and pro- (TNF-α and IL-6) and anti- (IL-10) inflammatory cytokine secretion (Figures 1D–F) of MAB-R and -S strains of various subspecies or genotypes [S-Abs smooth strains (S-Abs_S): *M. abscessus* type strain ATCC 19977 smooth strain, Asan 53040, and Asan 58582; S-Abs rough strains (S-Abs_R): *M. abscessus* type strain ATCC 19977 rough strain, Asan 52550 and Asan 58116; S-Mas type I-Smooth (S-Mas_I-S): type strain, Asan 15, Asan 51312, and Asan 51843; S-Mas type I-Rough (S-Mas_I-R): Asan 16, Asan 22, and Asan 34; and S-Mas type II-Rough (S-Mas_II-R): Asan 4, Asan 50594, and Asan 62188] in murine macrophage J774A.1 cells (1 × 10^6^ cells) as a function of the 10 multiplicity of infection (M.O.I.) (1 × 10^7^ bacteria) (Figures 1A,B). The survival of intracellular mycobacteria was examined with a colony-forming unit (CFU) assay. The results showed that irrespective of subspecies or genotypes, MAB-R strains formed significantly higher levels of CFUs than MAB-S strains in J774A.1 cells. A similar growth trend was also shown in a bone marrow-derived macrophage (BMDM) cells (Figure 1C). Of note, MAB-R and -S strains showed different intracellular growth kinetics. Briefly, there were no significant differences in intracellular growth between MAB-S and MAB-R infected cells at 2 h. MAB-R strains showed a marked increase in intracellular growth in BMDM as infection time increased (2 to 6 h and 6 to 24 h), but in contrast, MAB-S strains were almost invariable in intracellular growth in BMDM between 3 time points (2, 6, and 24 h). It suggests that the increased number of MAB-R strain in phagocytes may be due to its active replication within phagocytes.

To check the capacity of MAB-R and -S strains to induce the innate immune response, we measured cytokine production from supernatants of infected cells with 10 M.O.I at 24 h.p.i by enzyme-linked immunosorbent assay (ELISA). Both of the pro-inflammatory cytokines TNF-α and IL-6 were more induced in two different macrophages (J774A.1 cells and BMDMs) and in BMDCs infected with MAB-R strains than in those infected with MAB-S strains (Figures 1D–F). In addition, the anti-inflammatory cytokine IL-10 was also more induced in MAB-R strain-infected cells (Figures 1D–F). The expression of GPL, which is known to inhibit TLR2 signaling (29), is reduced in the MAB-R strains, and thus the expression of cytokines induced by MAB-R strains is increased, compared to MAB-S strains. Together, these results indicated that MAB-R strains showed increased intracellular growth and innate immune responses in murine macrophage than MAB-S strains, irrespective of the taxonomic status of subspecies or genotype. It also suggests MAB-R strains may promote enhanced airway inflammation compared to MAB-S strains.

### Increased Multiplication of MAB-R Strains in the Macrophage Phagosome Leads to Bacterial Phagosomal Escape After Phagosomal Rupture

**Figure 2:**
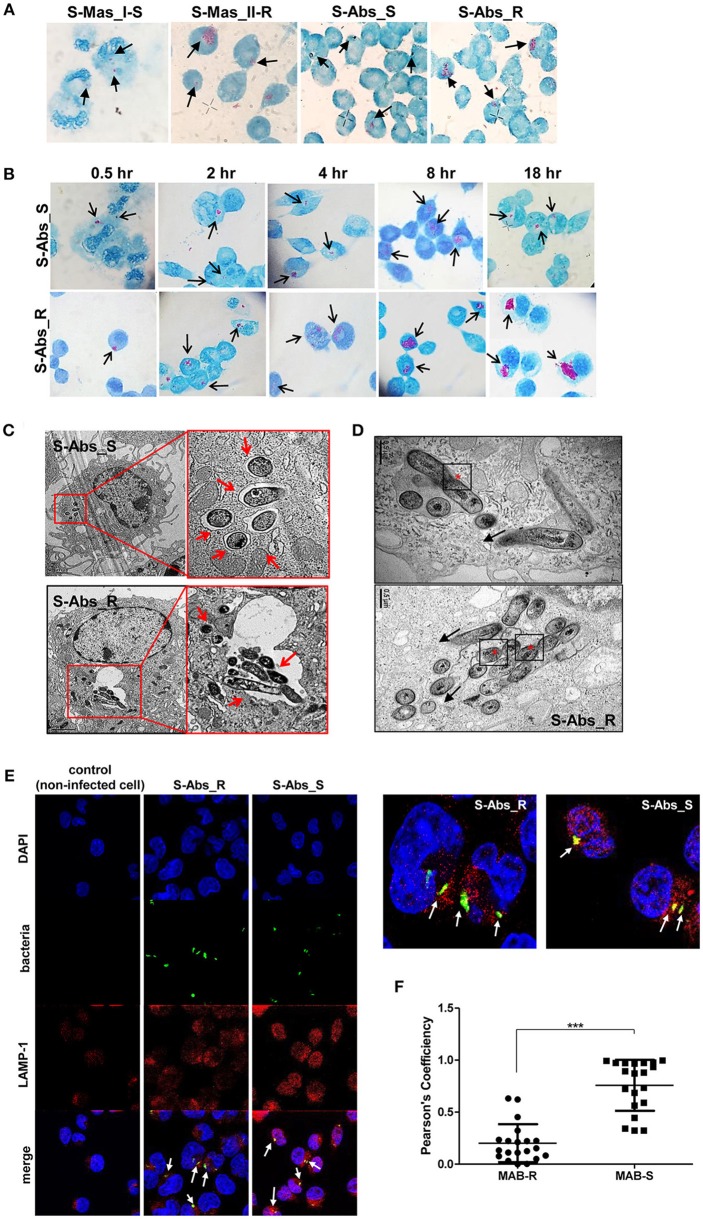
Morphological evidence of phagosomal escape and subsequent phagosomal rupture in MAB-R-infected murine macrophage. **(A)** AFB stain images of macrophages infected with MAB-R strains [S-Abs_R (*M. abscessus* type strain ATCC 19977 rough strain) and S-Mas_II-R (Asan 50594)] and MAB-S strains [S-Abs_S (*M. abscessus* type strain ATCC 19977 smooth strain) and S-Mas_I-S (Asan 51843)] at 10 M.O.I. for 24 h.p.i. The black arrow indicates mycobacteria (red-stained rods) in methylene blue-stained J774A.1 cells. **(B)** Comparison images of J774A.1 cells infected with 10 M.O.I. of S-Abs_R (*M. abscessus* type strain ATCC 19977 rough strain) and S-Abs_S (*M. abscessus* type strain ATCC 19977 smooth strain) at different time points (0.5, 2, 4, 8, and 18 h.p.i.). **(C)** Representative TEM images of BMDM cells infected with S-Abs_R (*M. abscessus* type strain ATCC 19977 rough strain) and S-Abs_S (*M. abscessus* type strain ATCC 19977 smooth strain) strains at 10 M.O.I. at 24 h.p.i. Phagosome morphology and magnified images are shown (red box region and red arrow). Bar indicates 0.5 or 2 μm. **(D)** Representative images of phagosome infected with an S-Abs_R (*M. abscessus* type strain ATCC 19977 rough strain). We marked a red asterisk to indicate bacterial replication in the phagosome. In this image, S-Abs_R was divided into two bacteria in the phagosome, reflecting active replication in the phagosome (red asterisk). S-Abs_R were also removed (black arrow) from the phagosome to the cytosol in macrophage. Bar indicates 0.5 μm. **(E)** J774A.1 cells were infected with 10 M.O.I of CFSE (green) stained bacteria [MAB-R (*M. abscessus* type strain ATCC 19977 rough strain) MAB-S (*M. abscessus* type strain ATCC 19977 smooth strain] for 24 h.p.i. and then stained with DAPI (blue) and LAMP-1 (red), representative images is shown. Non-infected J774A.1 cells were used as negative control. The white arrows showed co-localization of intracellular bacteria with LAMP-1 (yellow). **(F)** Quantification of Pearson's colocalization coefficient between MAB-R or -S and LAMP-1. The 20 bacteria randomly selected were analyzed and are a representative result out of two independent experiments. Results are means ± *SD* and ***P < 0.001 (Student's *t*-test).

To better understand the mechanism underlying the different growth kinetics during macrophage infections of MAB-R and -S strains (Figures 2A,B). We conducted transmission electron microscopy (TEM) and acid-fast bacillus (AFB) staining of infected murine macrophage. First, J774A.1 cells were infected with MAB-R and -S strains at an M.O.I. of 10 for 24 h (see Experimental procedures). After extensive washing to eliminate the residual extracellular bacteria, cells were fixed and processed for AFB staining. The results showed that while only a few MAB-S strains were phagocytized and confined into small phagosomes, several MAB-R strains were clustered in a few large phagosomes (Figure 2A). Furthermore, while the number of surviving MAB-S cells were almost always invariant at different time points [(0.5, 2, 4, 8, and 18 h.p.i. (hours post-infection)], the numbers of MAB-R cells within phagosomes appear to be increased with infection time, suggesting their active replication in phagosome (Figure 2B). This AFB staining data prompted us to hypothesize that MAB-R but not MAB-S, actively replicated in phagosomes in the initial phase of macrophage infection and that then bacterial overgrowth beyond the phagosome capacity could lead to phagosomal rupture. To further prove this hypothesis, BMDMs were infected with MAB-R and -S strains, and the intracellular localization of the bacteria was examined 24 h.p.i. by TEM. As previously reported (27), in MAB-S strains, single bacteria were located in a phagosome, and the outermost electron translucent zone (ETZ) (marked as a small arrow) (Figure 2C; Figure S1), which is a major part of the mycobacterial cell wall, was thick and poised all around the phagosome membrane. In contrast, MAB-R strains displayed a very thin ETZ, and several bacteria (up to more than 10 bacilli) were located in a larger phagosome (Figure 2C). We also found several pieces of TEM evidence supporting active multiplication through the binary fission of MAB-R in a phagosome of BMDM (Figure 2D; Figure S1), suggesting that the increased initial intracellular growth of MAB-R strains in macrophage may be due to the its active multiplication within the phagosome (bacterial replication indicated with an asterisk). In addition, our TEM-based study also showed that while MAB-S strains were primarily localized within membrane-bound vesicles within the phagosome, a few MAB-R strains could move from the phagosome into the cytosol of macrophages (marked as a black arrow) (Figure 2D; Figure S1). Furthermore, we also found that in some cases of MAB-R-infected macrophages, bacteria were released into the space, even with a concomitant rupture of the cell membrane (Figure 2D; Figure S1), which is consistent with a previous report that the bacterial cytosol access after phagosomal rupture could lead to cell-to-cell spreading via cell membrane ruptures (19). Furthermore, to prove cytosol access of MAB-R strain, we investigated colocalization of MAB-R strains and a late endosome marker (30), LAMP-1 via confocal microscopy. As shown in Figure 2E and Figures S1E,F, the colocalization of MAB-R with LAMP-1 was significantly lower than that of MAB-S (Figure 2F) at the 4 h.p.i and 24 h.p.i. Furthermore, colocalization intensity between MAB-R strains and LAMP-1 were decreased according to increase of infection time (4 to 24 h.p.i) (Figures S1E,F), strongly supporting our TEM based finding of phagosome escape of MAB-R strains.

**Figure 3:**
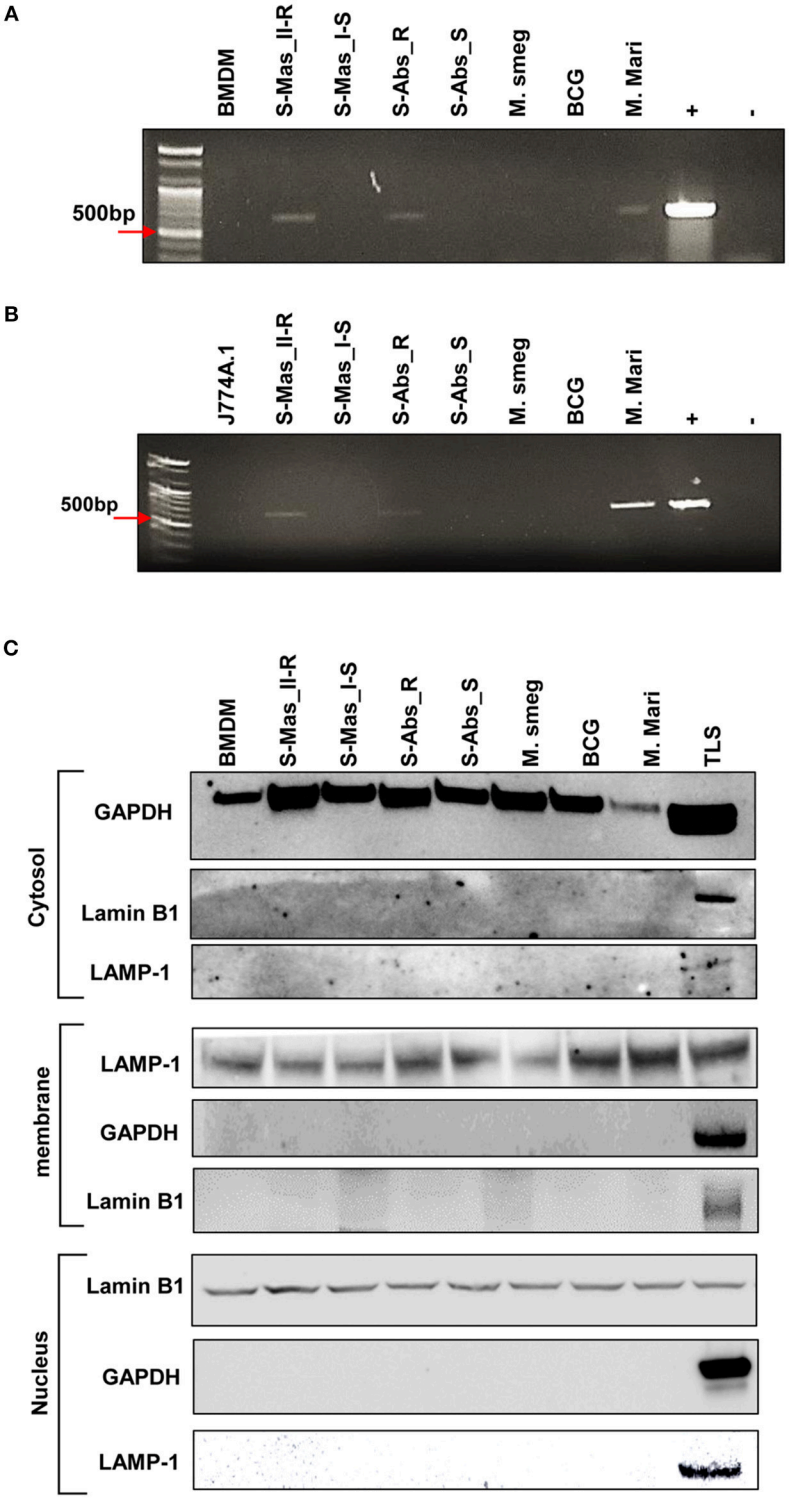
Detection of mycobacterial DNA from the cytosolic region of MAB-R-infected murine macrophages by PCR. **(A,B)** BMDMs **(A)** and J774A.1 cells **(B)** infected with MAB-R and -S strains [S-Mas_II-R (Asan 50594), S-Mas_I-S (Asan-51843) S-Abs_R (M. abscessus type strain ATCC 19977 rough strain), S-Abs_S (*M. abscessus* type strain ATCC 19977 smooth strain)], *M. smeg* (*M. smegmatis*), BCG (*M. bovis* BCG), and *M. mari* (*M. marinum*) at 37°C for 24 h. Cytosolic DNA (from cytosol fractionation) was extracted by the phenol-chloroform-isoamyl alcohol (PCI) method. Cytosolic DNA was detected by PCR amplification of the hsp65 (603 bp) gene (+, mycobacterial DNA; −, containing only primers). **(C)** Cytosolic cell fractions were isolated using the manufacturer's protocol for the Qproteome Cell Compartment Kit (QIAGEN). Western blotting was performed to confirm the separation of the cytoplasm and membrane, nucleus. To confirm this separation and exclude contamination during cell fraction isolation, GAPDH, LAMP-1 and Lamin B1 were used as a cytoplasmic marker, a phagosomal marker, and a nuclear marker, respectively. BMDM (non-infected cell), S-Mas_II-R: Asan 50594, S-Mas_I-S: Asan 51843, S-Abs_R: *M. abscessus* type strain ATCC 19977 rough strain, S-Abs_S: *M. abscessus* type strain ATCC 19977 smooth strain, *M. smeg*: *M. smegmatis*, BCG: *M. bovis* BCG, *M. mari*: *M. marinum*, and TLS: BMDM total lysate.

To further prove the phagosomal escape of MAB-R strains during infection, we evaluated the mycobacterial DNA in the nuclear and cytosol fractions in cytosol isolated from infected cells (Figure 3C) after phenol-chloroform isoamyl alcohol (PCI) DNA extraction (see Experimental procedures). As shown in Figure 3, we detected mycobacterial *hsp65* DNA in the cytosol fraction of macrophages with either one of two MAB-R strains [S-Abs_R (*M. abscessus* type strain ATCC 19977 rough strain) and S-Mas_II-R (Asan 50594)] or with *M. marinum*, which is known to be capable of phagosomal escape (21, 31) and served as a positive control; this DNA was not detected in this fraction from macrophages infected with either of two MAB-S strains [S-Abs_S (*M. abscessus* type strain ATCC 19977 smooth strain) and S-Mas_I-S (Asan 51843)], *M. smegmatis* or BCG (Figures 3A,B), further supporting our TEM finding of phagosomal escape by MAB-R strains. Together, our data indicated that MAB-R strains but not MAB-S strains lead phagosomal escape and subsequent phagosomal rupture, which may be due to active multiplication of MAB-R strains in macrophage phagosomes.

### Live MAB-R Stains Induce Type I IFN Secretion in an IRF3-Dependent Manner

**Figure 4:**
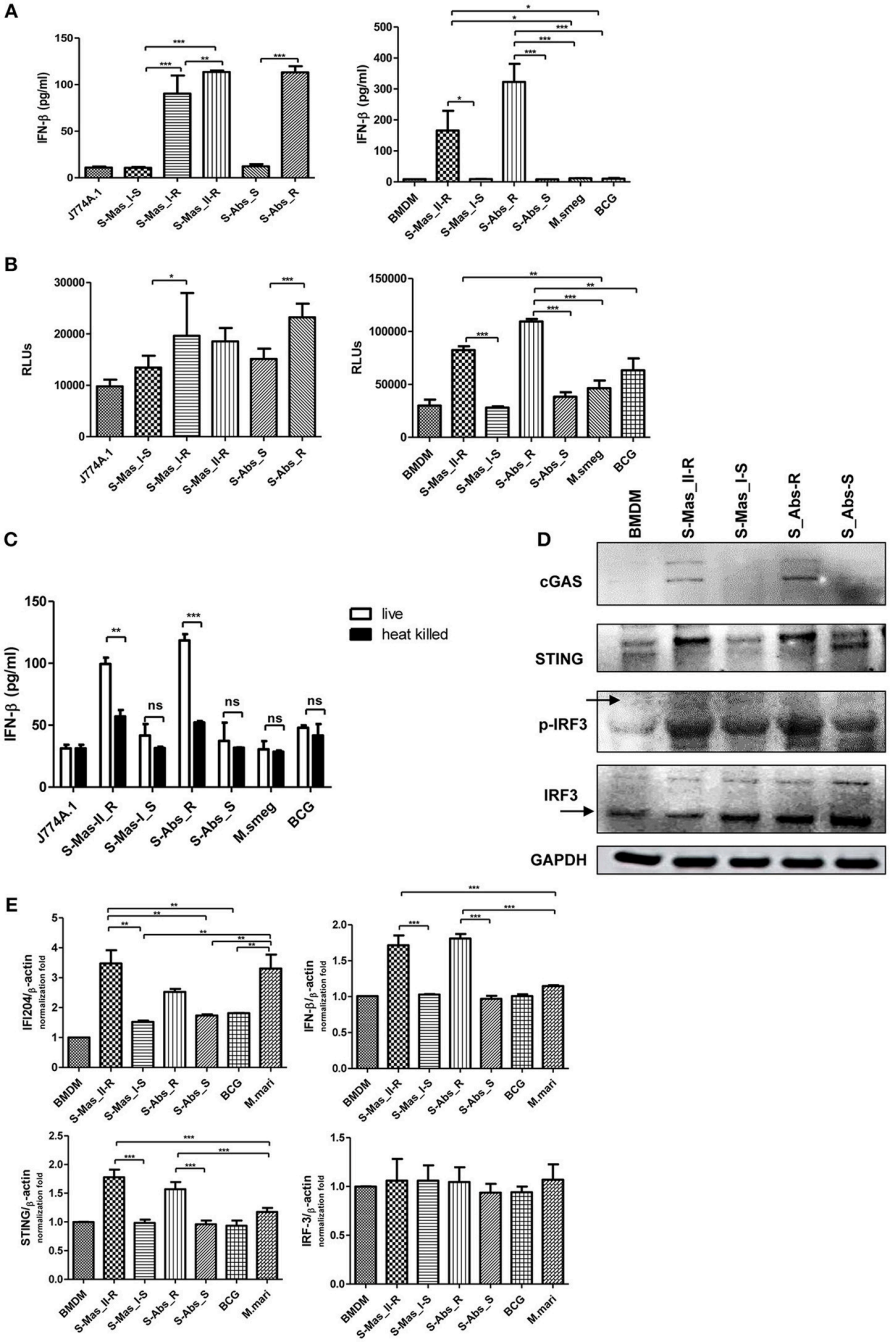
Live MAB-R strains induce Type I IFN secretion via the cGAS-STING-IRF3 axis. **(A,B)** Supernatants were harvested 24 h.p.i., and their Type I IFN levels were analyzed by IFN-β ELISA **(A)** and L929-ISRE luciferase bioassay **(B)** (32). The luciferase bioassay units are relative light units (RLUs). **(C)** J774A.1 cells were either uninfected (control) or infected with live or heat-killed (HK) bacteria, the supernatants of the infected cells were collected, and Type I IFN levels measured by IFN-β ELISA. **(D)** Western blot of IRF3, p-IRF3, cGAS, and STING induced by MAB-R [S-Abs_R (*M. abscessus* type strain ATCC 19977 rough strain)] and MAB-S strains [S-Abs_S (*M. abscessus* type strain ATCC 19977 smooth strain) and S-Mas_I-S (Asan 51843)], *M. smegmatis*, and BCG infection (10 M.O.I.) of BMDMs for 24 h. **(E)** qRT-PCR was used to measure the expression levels of IFI204, IFN-β, STING, and IRF3 mRNA in the BMDMs infected with rough strains [S-Abs_R (*M. abscessus* type strain ATCC 19977 rough strain)] and S-Mas_II-R (Asan 50594)] and smooth strains [S-Abs_S (*M. abscessus* type strain ATCC 19977 smooth strain) and S-Mas_I-S (Asan 51843)], *M. bovis* BCG, or *M. marinum* (10 M.O.I.). The expression levels represent relative fold changes based on the β-actin level. The results are representative of two independent experiments and represent means ± *SD. P*-values were determined by one-way ANOVA with Tukey's multiple comparison test **(A,B,E)** and paired Student's *t*-test **(C)** using GraphPad prism program: ns, non-significant; *P < 0.05; **P < 0.01, and ***P < 0.001.

The cytosol access of mycobacterial strains such as virulent *M. tuberculosis* or *M. marinum* in macrophage via phagosomal rupture induce Type I IFN secretion (3, 22–25). Therefore, to address whether MAB-R strains capable of cell escape in macrophage also causes IFN secretion, we checked the IFN-β secretion capacity of MAB-R strains in BMDM or J774A.1 cells by two different methods, a luciferase assay in L929 IFN reporter cells (32) and IFN-β ELISA. We found that irrespective of subspecies or genotypes, MAB-R strains always had a higher level of IFN-β secretions than MAB-S strains and other mycobacterial species known to be not capable of phagosomal escape in macrophage infections such as *M. smegmatis* or BCG in J774A.1 cells or BMDMs (Figure 4A). In L929 IFN reporter cells, a similar trend was shown, proving the validity of our findings (Figure 4B). Next, to check whether Type I IFN secretion of MAB-R strains depends on active multiplication, we compared IFN-β secretion levels between live and heat-killed (HK) MAB-R strains (Figure 4C). Only live, not HK, MAB-R strains induced IFN-β secretions, but MAB-S, *M. smegmatis* and BCG did not cause IFN-β secretion irrespective of whether they were HK or live cells, suggesting that phagosomal escape of MAB-R strains via active multiplication in macrophage phagosome may contribute to IFN-β secretions.

The mycobacterial DNA released into the macrophage cytosol has been reported to lead to IFN-β secretion via cGAS-STING-IRF3 axis signaling (3, 22, 23). To address this issue, we investigated the IRF3 activation of MAB-R strains by western blotting (Figure 4D). Our data showed that only two MAB-R strains, S-Abs_R (*M. abscessus* type strain ATCC 19977 rough strain), and S-Mas_II-R (Asan 50594), induced IRF-3 activation in infected BMDMs. We also found increased levels of the activated forms of STING (33–35 kDa) and cGAS (62 kDa) in these two MAB-R strains, consistent with the previous result that cytosolically exposed DNA induced Type I IFN secretion via the cGAS -STING axis (3, 33). The similar trend was also found in infected BMDCs (Figure S2). Next, we examined the activation capacity of Type I IFN-related gene expression in different mycobacterial strains by quantitative RT-PCR 24 h.p.i. of BMDM (Figure 4E). Our data showed that transcription of the IFI204, IFNβ, STING, gene was induced via infection of only 3 strains, two strains of MAB-R [S-Abs_R (*M. abscessus* type strain ATCC 19977 rough strain), and S-Mas_II-R (Asan 50594)] and *M. marinum*, and not by infection of other mycobacterial strains ([S-Abs_S (*M. abscessus* type strain ATCC 19977 smooth strain) and S-Mas_I-S (Asan 51843)] and *M. bovis* BCG), further proving the enhanced Type I IFN signaling by MAB-R strains. But, there were no significant differences in IRF3 transcription levels between MAB-R strains and other mycobacteria (Figure 4E), suggesting enhanced production Type I IFN of MAB-R strains may be due to IRF3 activation, rather than enhanced expression of IRF3. Together, our data suggested that cytosolic DNA of MAB-R strains followed by active multiplication-mediated phagosomal rupture induces Type I IFN secretion via cGAS-STING-IRF3 axis signaling.

### Live MAB-R Stains Enhanced Cell Death in Murine Macrophage

**Figure 5:**
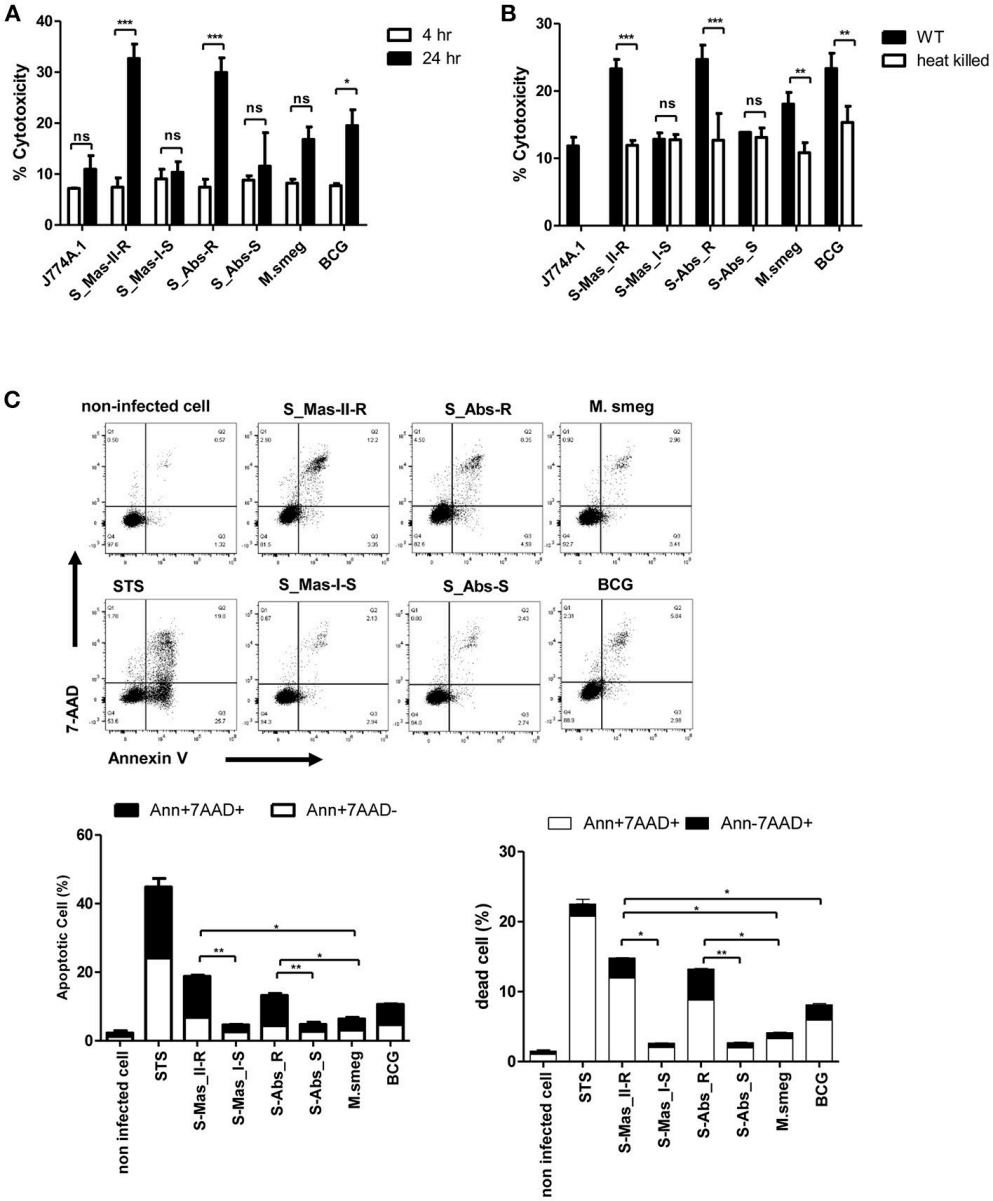
Live MAB-R stains enhance apoptotic cell death in murine macrophage. **(A)** To evaluate cytotoxic effects, cells were infected with S-Mas_II-R (Asan 50594), S-Mas_I-S (Asan 51843), S-Abs_R (*M. abscessus* type strain ATCC 19977 rough strain), S-Abs_S (*M. abscessus* type strain ATCC 19977 smooth strain), *M. smegmatis*, and *M. bovis* BCG (10 M.O.I.) in BMDM for 4 and 24 h. Cytotoxicity was quantitated by measurement of LDH activity in the culture supernatants using a CytoTox 96 assay kit (Promega) according to the manufacturer's protocol. Error bars represent the standard deviation of at least three independent experiments (*P < 0.05, **P < 0.01, ***P < 0.001; one-way ANOVA with Tukey's correction). **(B)** J774A.1 cells were either uninfected (control) or infected with live or heat-killed (HK) bacteria, supernatants of the infected cells were collected and cytotoxicity was measured by LDH assay. **(C)** J774A.1 cells were pre-treated with 100 nM staurosporine (apoptosis inducer) used as a positive control and infected (M.O.I. 10) with the indicated bacterial strains for 24 h.p.i. Cells were stained with Annexin V and 7-AAD, after then analyzed by flow cytometry (FACS LSRFortessa X-20). The results are representative of two independent experiments and represent means ± *SD. P*-values were determined by the Student's *t*-test using GraphPad prism program: ns, non-significant; *P < 0.05; **P < 0.01, and ***P < 0.001.

Phagosomal rupture in macrophage by virulent mycobacteria has been reported to induce cell death followed by cell-to-cell spread of cytosolic bacteria into neighboring cells via cell membrane rupture (24, 25). To address whether MAB-R strains capable of inducing phagosomal rupture also enhance the cell death of infected macrophages, we first examined the cytotoxicity of BMDMs infected with various mycobacteria by lactate dehydrogenase (LDH) assay for 4 and 24 h.p.i. As shown in Figure 5A, there were no significant differences in cytotoxicity between MAB-S and MAB-R infected cells in early infection time (4 h.p.i), however, MAB-R strains showed more cytotoxicity in 24 h.p.i. than other mycobacterial strains. Next, to address whether cell death induced by MAB-R is due to its active replication, we compared cell death-inducing capacity of various live and HK mycobacteria in infected J774A.1 cells (Figure 5B). Our data showed that only live MAB-R strains led to significantly greater cell cytotoxicity than HK bacteria, although in other mycobacterial strains (MAB-S, *M. smegmatis* or BCG), no significant difference in cell death-inducing capacity between live and HK bacteria was found, suggesting that the active multiplication of MAB-R strains may contribute to enhanced cell cytotoxicity.

Next, to determine what type of cell death is induced by infection of MAB-R strains, we compared features of induced cell death between various mycobacteria, including MAB-R strains. FACS analysis was conducted by using double staining with both annexin V (for apoptotic cell death) and 7-AAD (for necrotic cell death). Our data showed that two MAB-R strains more induced both apoptotic and necrotic cell death than other mycobacterial strains in a time dependent manner (Figure 5C; Figure S3A). In contrast, macrophage infected by two MAB-S strains appear to be resistant to both apoptotic and necrotic cell death even more than those infected by *M. smegmatis* in all the infected time points than MAB-R strains (Figure 5C; Figure S3A).

Furthermore, we also found that live MAB-R strains but not HK strains induced apoptotic and necrotic cell deaths (Figure S3B). Together, our data indicate that active multiplication of MAB-R strains contribute to the cell death (including apoptosis and necrosis) of infected murine macrophage.

### The Caspase Dependent Apoptotic Cell Death by MAB-R Strains Contribute Into Their Cell-To-Cell Spread

**Figure 6:**
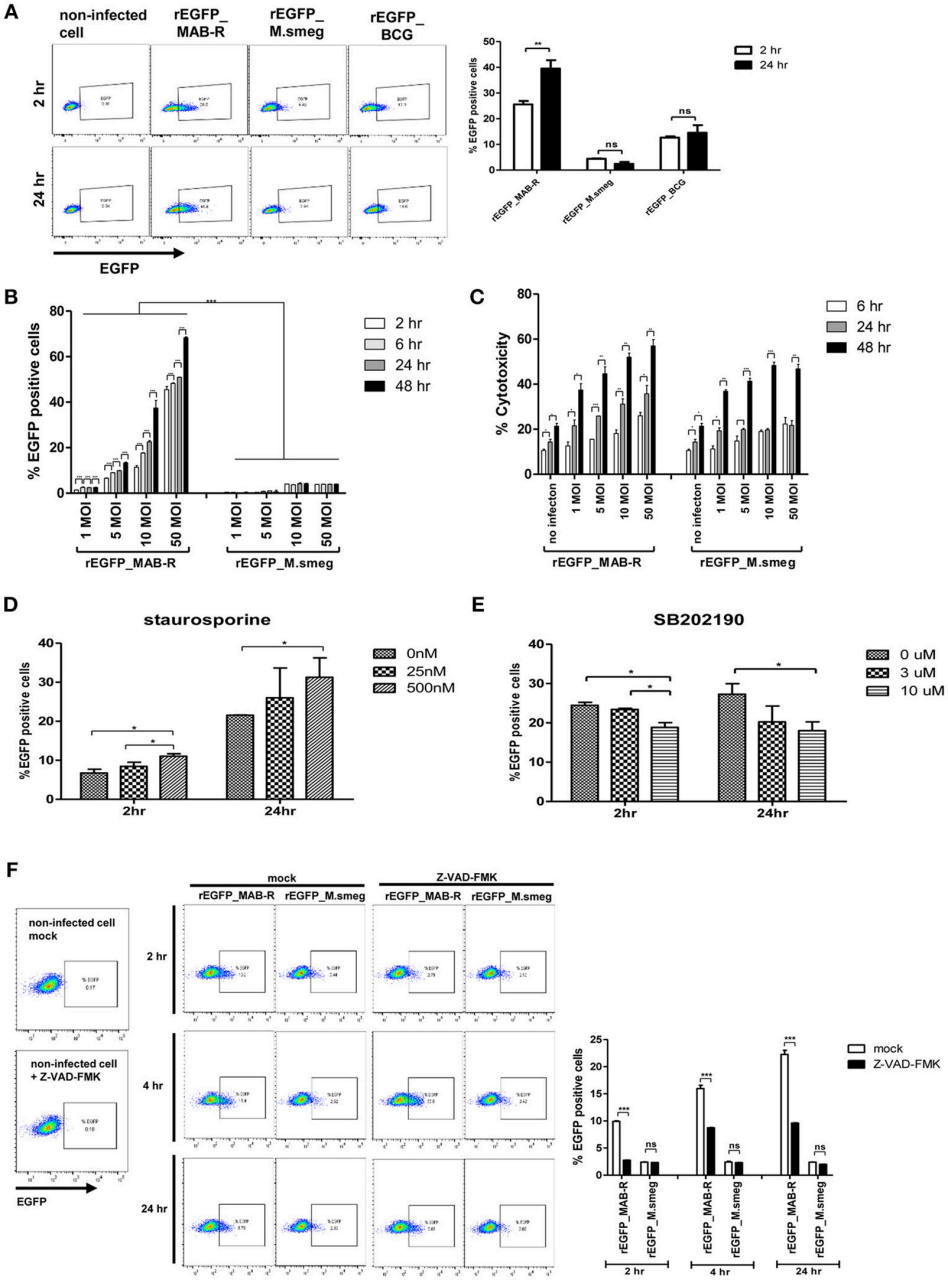
Enhanced apoptotic cell death by a MAB-R strain contributes to its cell-to-cell spread. **(A)** BMDMs infected with EGFP-expressing bacteria [EGFP recombinants: rEGFP_MAB-R (Asan 50594), rEGFP_M.smeg (*M. smegmatis*), rEGFP_BCG (*M. bovis BCG*)] for 0 and 24 h. EGFP-positive cells were analyzed by flow cytometry. **(B,C)** J774A.1 cells were infected with rEGFP_MAB-R (Asan 50594) and rEGFP_M.smeg (*M. smegmatis*), and analysis was performed on data at different time points (2, 6, 24, and 48 h) or M.O.I.s (1, 5, 10, and 50). **(B)** The percentage of EGFP-positive cells was evaluated by flow cytometry (FACScalibur). **(C)** Cytotoxicity was quantitated by measurement of LDH activity in the infected cell supernatants. **(D,E)** J774A.1 cells were pretreated with staurosporine (apoptosis inducer) or SB202190 (p38 MAPK inhibitor) and infected with MAB-R [rEGFP_MAB-R (Asan 50594)] (10 M.O.I.), and the percentage of EGFP-positive cells was measured by flow cytometry (FACScalibur). **(F)** J774A.1 cells were pre-treated with 10 uM Z-VAD-FMK (pan-caspase inhibitor) for 2 to 24 hr and infected with rEGFP_MAB-R and rEGFP_M.smeg (10 M.O.I) for and the percentage of EGFP-positive cells was measured by flow cytometry (FACScalibur). The results are representative of two independent experiments and represent means ± *SD. P*-values were determined by the Student's *t*-test using GraphPad prism program: ns, non-significant; *P < 0.05; **P < 0.01, and ***P < 0.001.

Previously, cell death by virulent mycobacterial strains such as *M. tuberculosis* H37Rv has been reported to contribute to bacteria cell-to-cell spreading, thus leading to increased bacterial growth (25). Therefore, to address whether cell death by MAB-R strains also contributes to enhanced intracellular bacterial growth via cell-to-cell spreading, we first compared the cell-to-cell spreading capacity of EGFP-expressing recombinant strains of MAB-R (Asan 50594), *M. smegmatis* and BCG infecting J774A.1 cells for 24 h.p.i. The cell spreading capacity was analyzed via FACS analysis as previously reported (25). We compared cell-to-cell spread FACS analysis by making recombinant bacteria expressing EGFP (Figure S4A in Supplementary Material). Our data showed that the MAB-R strain induced greater cell-to-cell spreading than *M. smegmatis* or BCG, reflecting the above finding that MAB-R strains induce cell death (Figure 6A). Next, to analyze the time kinetics and M.O.I. dependency in the cell spreading of the MAB-R strain and *M. smegmatis*, we investigated bacterial cell spreading in J774A.1 cells infected with the MAB-R or *M. smegmatis* strain at different time points (2, 6, 24, and 48 h) with different M.O.I. (1, 5, 10, and 50). We found that the cell spreading of MAB-R, but not *M. smegmatis* was gradually increased in a time and in an M.O.I dependent manner (Figure 6B and Figure S4B in Supplementary Material). In MAB-R strains, cytotoxicity of infected cells was also increased in a time and in an M.O.I dependent manner. In *M. smegmatis*, significant increase of cytotoxicity was also found between 6 and 24 h.p.i (Figure 6C). We found that the treatment of MAB-R-infected J774A.1 cells with staurosporine enhanced cell-to-cell spreading of bacteria in a dose-dependent manner (Figures 6D,E; Figures S4C,D). In contrast, the treatment with SB202190 decreased the cell-to-cell spreading of bacteria in a dose-dependent manner (Figure 6E; Figure S4D). Also, the cell-to-cell spreading induced by MAB-R strains was also decreased in the presence of pan-caspase inhibitor (Z-VAD-FMK) in a time dependent manner (Figure 6F), suggesting that apoptotic cell death may in part contributes to the macrophage cell-to-cell spreading of MAB-R strains.

### Enhanced Cell-To-Cell Spreading of MAB-R Strains Is Due to an IFNAR1-Dependent Pathway

**Figure 7:**
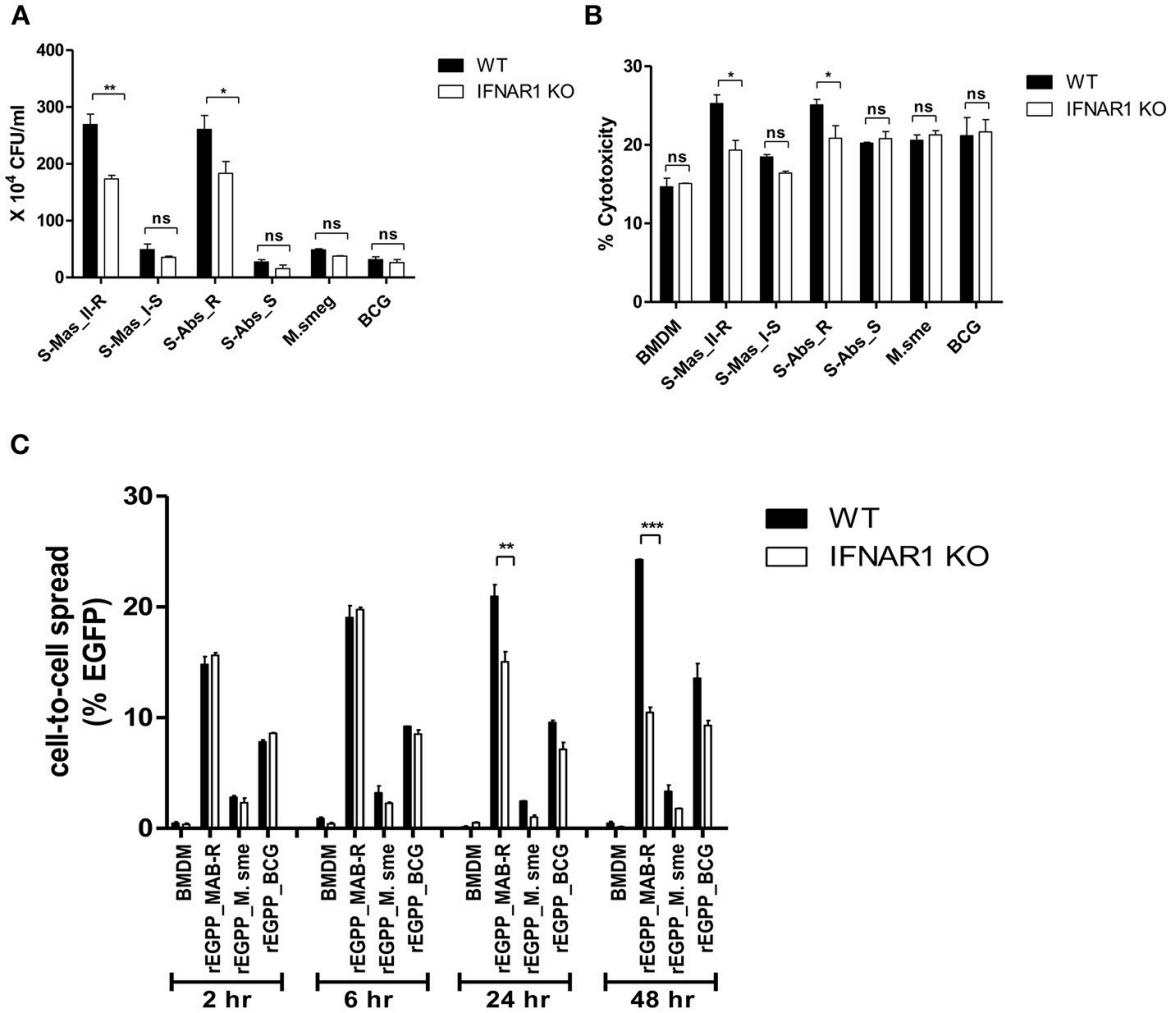
The cell-to-cell spread in MAB-R infection depends on Type I IFN signaling. **(A,B)** BMDMs (WT and IFNAR1 KO mice) infected with rough strains R [S-Abs_R (*M. abscessus* type strain ATCC 19977 rough strain)] and smooth strains [S-Abs_S (*M. abscessus* type strain ATCC 19977 smooth strain) and S-Mas_I-S (Asan 51843)], *M. smegmatis*, or *M. bovis* BCG (10 M.O.I) for 24 h. Infected cell lysate was used for CFU assays. Supernatants were collected and cytotoxicity was measured by LDH assay. **(C)** BMDMs (WT and IFNAR1 KO mice) infected with EGFP-expressing bacteria [rEGFP_MAB-R (Asan 50594), rEGFP_M.smeg, rEGFP_BCG] (10 M.O.I) measured at different time points (2, 6, 24, and 48 h). Infected cells were collected and the percentage of EGFP-positive cells was evaluated by flow cytometry (FACScalibur). The results are representative of two independent experiments and represent means ± *SD. P*-values were determined by the Student's *t*-test using GraphPad prism program: ns, non-significant; *P < 0.05; **P < 0.01, and ***P < 0.001.

Type I IFN signaling induces apoptosis in the splenocytes from mice and contributes to its virulence via cell-to-cell spreading in *Listeria monocytogenes* infections (34, 35). Therefore, we sought to explore whether Type I IFN induced by MAB-R infection could also contribute to bacterial cell-to-cell spreading via enhanced cell death. First, we compared the Type I IFN-dependent survival capacity in macrophage infected with various mycobacteria. To this end, we infected BMDMs from wild or IFNAR1 KO mice with mycobacteria and then compared their intracellular survival capacities. The result showed that only two MAB-R strains (not MAB-S, *M. smegmatis*, or BCG) led to significantly lower survival capacity in infected BMDM cells from IFNAR1 KO mice than in infected BMDMs from wild mice (Figure 7A), suggesting that Type I IFN signaling may contribute to the enhanced intracellular growth in MAB-R-infected macrophages.

Next, to determine the Type I IFN dependency of MAB-R-induced cytotoxicity, we infected various mycobacterial strains into BMDMs from wild-type or IFNAR1 KO mice with 10 M.O.I. and examined them 24 h.p.i. As a result, we found that only two MAB-R strains led to significantly less cell cytotoxicity in BMDMs from IFNAR1 KO than those from wild mice; however, there were no significant changes in other mycobacteria, including two MAB-S strains, *M. smegmatis* and BCG (Figure 7B), suggesting that the Type I IFN dependency of MAB-R strains induces cell cytotoxicity.

Next, to check whether Type I IFN dependency in MAB-R strains induced cell-to-cell spread, we compared the cell-to-cell spreading capacity of EGFP-expressing recombinant strains of MAB-R [rEGFP_MAB-R (Asan 50594)] strain, *M. smegmatis* (rEGFP_M.smeg), and *M. bovis* BCG (rEGFP_BCG) after their infection into BMDMs from wild mice or IFNAR1 KO mice. We found that MAB-R but not *M. smegmatis* and BCG led to significantly less cell-to-cell spreading in BMDMs of IFNAR1 KO mice at the 24 and 48 h.p.i. than in BMDMs of wild type (Figure 7C; Figure S5), suggesting a partial contribution of Type I IFN signaling to MAB-R-induced cell-to-cell spreading. Together, our data demonstrated that apoptotic cell death by MAB-R strains contributes to enhanced intracellular bacterial growth via providing a new niche for bacterial growth resulting from cell-to-cell spread. In addition, Type I IFN signaling induced by MAB-R infection partially contributes to their cell spreading.

## Discussion

We have previously reported that the S-Mas type II genotype (rough type) leads to the production of a higher level of CFUs and TNF-α secretion from human monocytes than the Type I genotype (smooth type) (26). It was recently reported that highly virulent clinical MAB strains lead to more cell death than non-virulent strains (2). Furthermore, virulent *M. tuberculosis* strains have been reported to survive better than attenuated strains because of their cell-to-cell spreading and bacterial cytosol access via ESX-1-derived active phagosomal rupture (20–22). In this process, bacterial DNA exposed in the cytosol induces Type I IFN signaling, resulting in apoptotic cell death (22–25). These previous findings prompt us to hypothesize that the higher pathogenic potential in MAB-R strains vs. MAB-S strains may also be due to mechanisms similar to those in virulent *M. tuberculosis*. Indeed, in the present study, we found that irrespective of different subspecies or genotypes, virulent MAB-R strains have cytosolic stages that are followed by phagosomal replication and rupture in the course of infecting murine macrophages (Figure 2; Figure S1), consequently leading to the induction of Type I IFN signaling (Figure 4) and cell death-mediated cell-to-cell spreading (Figures 5–7).

Unlike virulent *M. tuberculosis* or *M. marinum* strains harboring the ESX-1 locus (20–22) or some specialized intracellular bacteria such as *Shigella flexneri* (36), *Listeria monocytogenes* (37) or *Francisella tularensis* (38) that can actively destroy the phagosome membrane, MAB strains without an ESX-1 ortholog in their genome (28) have to utilize an alternative strategy for bacterial cytosol access. Recently, the orthologous ESX-4 locus in *M. abscessus* genome has been reported to be capable of elicit phagosome membrane rupture and be essential for the intracellular survival of *M. abscessus* in amoeba or macrophage, strongly supporting the possibility that ESX-4 may operate as a surrogate for ESX-1 in *M. tuberculosis* (39). Indeed, we found via TEM and AFB staining that abrupt increases in the intracellular growth of MAB-R strain during the initial phase of infecting macrophages (6 to 24 h.p.i.) was due to their active multiplication in phagosome, leading to phagosomal rupture via their overgrowth beyond phagosome capacity (Figure 2; Figures S1B,D), which could consequently lead to their escape into the cytosol. Our TEM and AFB staining data indicated that even in some MAB-R-infected cells, bacterial release into the extracellular medium from destroyed cell membranes occurred (Figure 2B; Figures S1C,D), suggesting a possible link between the phagosomal escape of MAB-R strains by phagosomal rupture to cell death-mediated bacterial cell-to-cell spreading. The bacterial escape into the cytosol via overgrowth in phagosomes that was exhibited by MAB-R strains has not been previously reported. However, whether ESX-4 locus of *M. abscessus* could contribute into active replication in phagosome, phagosome escape, and intracellular survival of MAB-R strains in infected macrophages should be elucidated in the future.

In contrast to MAB-R strains, the MAB-S strains rarely increased their intracellular growth in the initial stage of infection (Figure 1C), suggesting a lack of growth in the phagosome, which is consistent with our TEM data showing a single bacterium per vacuole in most vacuoles and no evidence of phagosomal rupture (Figure 2C; Figure S1A). The disparity in phagosomal rupture-inducing capacity between MAB-R and -S types can lead to distinct damage-associated molecular pattern (DAMP) responses between the two groups. Indeed, we found that MAB-S strains produced lower amounts of proinflammatory cytokines such as TNF-α and IL-6 than MAB-R strains in infections of murine macrophage or dendritic cells (Figures 1D–F). Furthermore, the contrast in produced cytokines between live and HK bacteria was less pronounced in MAB-S-infected macrophage than in MAB-R-infected macrophage (Figure S6). It has been reported that MAB-S infection is more prevalent during initial infection from environments, but after infection, a morphotype change from the MAB-S type into the MAB-R type frequently follows (2, 17, 18). Therefore, in macrophage infections with both morphotypes, MAB-S strains may enable the initial infection and the colonization into macrophage by minimally evoking host immune responses like a latent infection of *M. tuberculosis*, and their transition into the MAB-R type could broaden its niche for intracellular survival in the late phase of infection at the expense of evoking host immune responses.

In addition to TEM-based evidence, there are two other findings (biochemical evidence and PCR-based detection) supporting the presence of cytosol stages of MAB-R strains in infected macrophage. First, we also found that irrespective of the types of their subspecies (S-Abs or S-Mas) or genotypes (Type I or Type II of S-Mas), MAB-R strains but not MAB-S strains induced IFN-β secretion (Figure 4A), ISRE-dependent luciferase activity (Figure 4B) and the transcriptional expression of downstream interferon-related genes (Figure 4E) in an IRF3-dependent manner in infected BMDMs or BMDCs, further providing clear biochemical evidence for their cytosol access. Moreover, our finding that only their live strains and not HK strains induce IFN-β secretion (Figure 4C) proves that their induction of Type I IFN may be due to DAMP (cytosolic bacterial DNAs)-dependent intracellular signaling rather than extracellular signaling by membrane-associated pattern recognition receptors (PRRs) such as TLR3 or TLR9. Indeed, we also found an increase in activated STING proteins in MAB-R-infected BMDM or BMDCs (Figure 4D; Figure S2), suggesting the direct sensing of cytosolic bacterial DNA. For the second piece of evidence supporting the cytosol stages of MAB-R strains, we directly detected the presence of cytosolic MAB DNA by a PCR method targeting mycobacterial *hsp65* genes in MAB-R-infected murine macrophages (J774A.1 cells and BMDMs) (Figures 3A,B).

**Figure 8:**
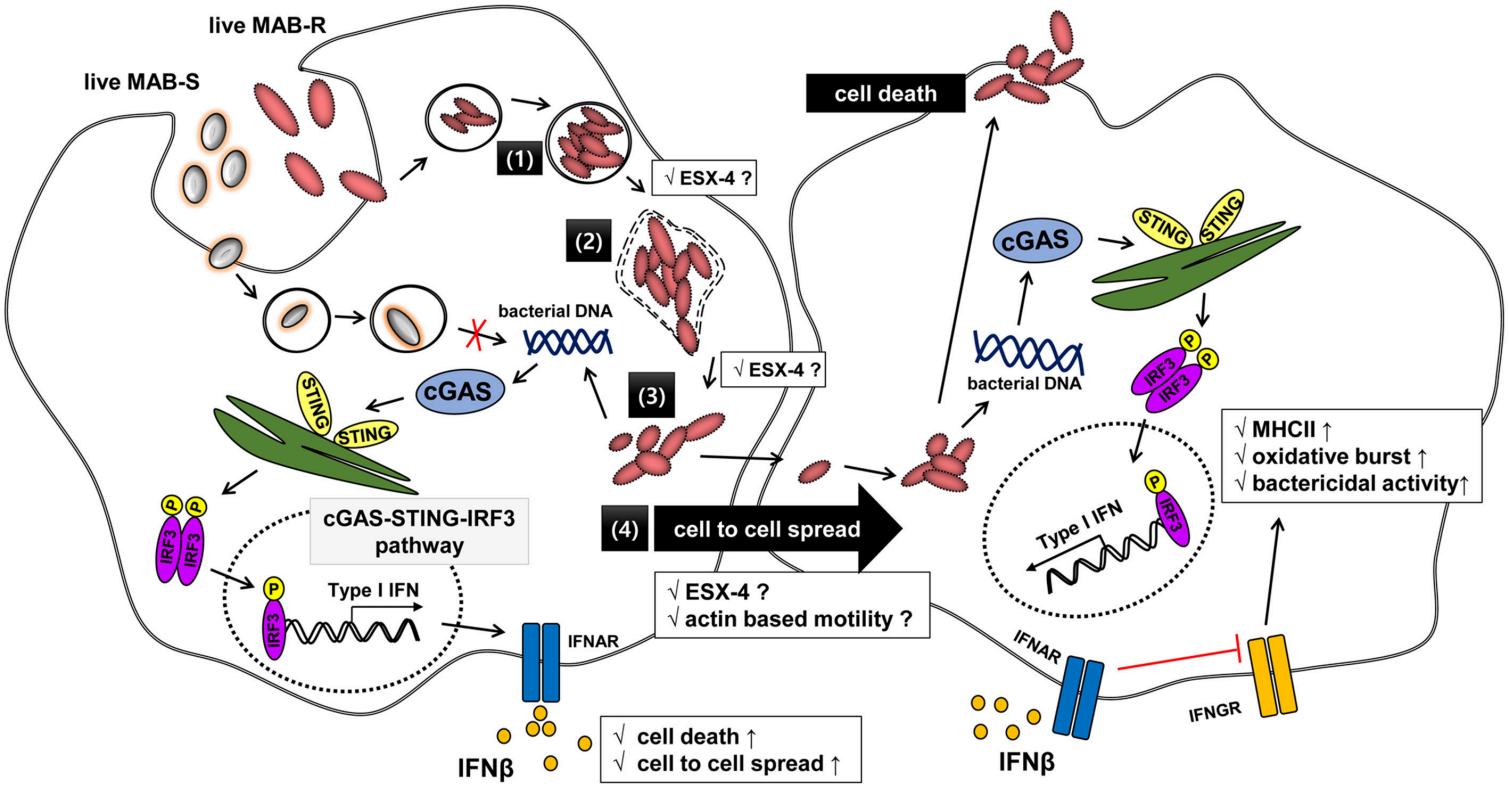
Schematic representation showing phagosomal rupture-mediated phagosomal escape of MAB-R in infected murine macrophage, leading to Type I INF production via the cGAS-STING-IRF3 axis. Live *M. abscessus* (MAB-R and -S) move into macrophage cells by phagocytosis. However, different life styles within macrophages were found between MAB-R and MAB-S strains. MAB-S strains remain silent in the phagosome and cannot lead to phagosomal rupture mediated escape to cytosol. So, MAB-S strains also cannot activate Type 1 IFN production via the cGAS-STING-IRF3 pathway. However, MAB-R can replicate in the phagosome (1) and cause phagosomal rupture (2). MAB-R can escape to the cytosol (3), which produce Type I IFN (IFNβ) via the cGAS-STING-IRF3 pathway (4). Furthermore, MAB-R can move to adjacent cells by Type I IFN receptor-dependent cell-to-cell spreading. ESX-4 locus, which has been recently reported to be related to phagosomal rupture (39), may contribute into replication in phagosome (1), phagosome rupture (2), and escape into cytosol of MAB-R (3). Finally, secreted IFN-β by MAB-R infections could also contribute into bacteria proliferation by interfering with IFN-γ signaling pathway (48).

Type I IFN plays a pivotal role in immunity and inflammation in an autocrine or a paracrine manner via interactions with its receptor, IFNAR1, which is ubiquitously expressed in a variety of cells (40, 41). Although the host-protective functions of Type I IFNs have been well-described in viral infections, they are also generally considered to have a detrimental effect on diseases caused by intracellular bacteria, such as *M. tuberculosis* (42–44). For example, in humans, a comparable IFN-inducible gene signature was observed in the blood of TB patients as well as in 10–25% of latently infected individuals, suggesting that the whole-blood IFN signature could be useful in identifying active TB disease (45). Furthermore, a recent *in vivo* mice study proved that coinfection with a virulent *M. tuberculosis* infection could increase the severity of other viral infections such as influenza in a Type I IFN signaling-dependent manner (46). Since avirulent or saprophytic mycobacteria cannot induce type I IFNs, it is hypothesized that Type I IFN production is associated with mycobacterial virulence and increased host susceptibility (47). Furthermore, Induction of Type I IFN has also been reported to lead to proliferation of intracellular bacteria such as L. monocytogenes within macrophage via inhibiting macrophage activation by IFN-γ (48) (Figure 8). Indeed, our findings of enhanced Type I IFN production in virulent strains (MAB-R) strains but not in the less virulent type (MAB-S) (Figures 7A,B) also support the above hypothesis. Therefore, chronic stimulation of the Type I IFN signaling pathway, induced by MAB-R infection, could compromise host-defense immune responses against its infection and affect disease progression by other infections co-infecting with MAB-R, such as *M. tuberculosis* or influenza.

Apoptotic cell death via phagosomal rupture in pathogenic mycobacterial infection can lead to cell-to-cell spreading, which contributes to their survival in macrophage (25, 31, 49, 50). A recent study showed that Type I IFN signaling induced by *Listeria monocytogenes* infections contributes to their virulence via cell-to-cell spreading (35). Indeed, in this study, we proved that enhanced apoptotic cell death by MAB-R strains leads to cell-to-cell spreading in an IFNAR1-dependent manner (Figure 7C; Figure S5), suggesting that Type I IFN in MAB-R strains contributes to their enhanced virulence via providing a niche for their survival within macrophage (Figure 7A).

Recently, it has been reported that Type I IFN can promote cell to cell spreads of *L. monocytogenes* in *in vitro* macrophage or in livers of infected mice, which is mediated by formation of actin comet tails via IFNRR1 (35). So it is tempting to speculate that Type I IFN dependent cell-to-cell spread of MAB-R strains as shown in this study (Figure 7A), may also be due to the similar mechanism as shown in *L. monocytogenes* (Figure 8). However, issue regarding actin based motility or actin comet formation of MAB-R strains for cell-to-cell spread remain to be addressed in the future.

In conclusion, our data indicated that the transition of MAB-S variants into R variants increases their virulence via enhanced Type I IFN signaling, which leads to enhanced survival in infected macrophage via their cell death-mediated cell-to-cell spreading. These results provide not only a novel insight into different levels of virulence between MAB-R and S variants but also a hint into their treatment strategy (Figure 8).

## Materials and Methods

### Mice

Male C57BL/6 mice and C57BL/6 IFNAR KO mice (~25 g, 6 weeks old) were purchased from Orient-Bio (Seongnam, South Korea). All mice were housed and maintained in a specific pathogen-free facility at Seoul National University College of Medicine. All mice were used for experiments at 8~12 weeks of age.

### Cell Culture

J774A.1 (murine macrophage) and L929 cells (mouse fibroblast, CCL-1) were maintained in Dulbecco's modified Eagle's medium (DMEM) containing 10% fetal bovine serum and penicillin/streptomycin (100 U/ml) and kept at 37°C and 5% CO_2_ in humid incubator.

### Preparation of Mouse Bone Marrow-Derived Macrophages (BMDMs) and Mouse Bone Marrow-Derived Dendritic Cells (BMDCs)

BMDMs and BMDCs were generated from the bone marrow (BM) of 8 to 12-week-old C57BL/6J mice. BM cells were obtained from femurs and tibia of mice and flushed out with serum-free DMEM media (BMDM; Gibco) or serum-free Iscove's modified Eagle's medium (BMDC, IMDM; Gibco Invitrogen). The single-cell suspension was then filtered through a nylon cell strainer (70-μm Nylon mesh; BD Pharmingen, Franklin Lakes, NJ, USA) and washed twice with HBSS media. The cells were pelleted by centrifugation (350 × g, 5 min), and the supernatant was aspirated. The pellet was resuspended with red blood cell lysis buffer (Sigma) and incubated for 3 min at room temperature; the reaction was stopped by diluting the Lysis Buffer with V/V ml of serum-containing DMEM (BMDMs) or complete IMDM (BMDCs). The cells were spun (350 × *g*, 5 min), and the supernatant was discarded. BMDMs were differentiated from bone marrow precursors cultured for 4 days in complete DMEM containing 10% FBS, 1% sodium pyruvate, 4 mM glutamine, and 1% penicillin-streptomycin (100 units/ml) supplemented with 10% L929 cell-conditioned media. Total BM cells were cultured in microbiological 12- or 24-well plates (Sterilin) and kept at 37°C and in 5% CO_2_ in a humid incubator. BMDCs were differentiated with complete IMDM supplemented with 10% FBS (Gibco, Invitrogen), recombinant mouse GM-CSF (20 ng/ml, PeproTech, Rocky Hill, NJ, USA) and mouse IL-4 (20 ng/ml; PeproTech), penicillin (100 units/ml; Gibco Invitrogen), streptomycin (100 μg/ml; Gibco Invitrogen), gentamicin (50 μg/ml; Gibco Invitrogen), L-glutamine (2 mM; Gibco Invitrogen), and β-mercaptoethanol (50 nM; Gibco Invitrogen) and seeded at a concentration of 1 × 10^6^ cells per well in a 24-well plate in a final volume of 2 ml of complete IMDM. Half of the medium was replaced every other day with an equal volume of complete IMDM for 6 days. All the cells expressing CD11C^+^ in the different wells were isolated using appropriate magnetic-activated cell sorting (MACS) kits according to the manufacturer's instruction (Miltenyi Biotec). CD11c^+^ cells were collected and used in all experiments.

### Bacterial Strains and Growth Conditions

The mycobacterial strains used in this study are as follows: 5 type strains of *Mycobacterium abscessus* (ATCC 19977 ^T^ rough and smooth type), *Mycobacterium abscessus* subsp. *massiliense* (CIP 108297^T^), *Mycobacterium smegmatis* ATCC 19420^T^, *Mycobacterium bovis* BCG Tokyo strain (BCG), and *Mycobacterium marinum* JCM 17638^T^. Thirteen clinical isolates of *Mycobacterium abscessus* smooth (S-Abs_S; Asan 53040 and Asan 58582) and rough (As S-Abs_R; Asan 52550 and Asan 58116), *Mycobacterium abscessus* subsp. *massiliense* Type I smooth (S-Mas_I-S; Asan 15 and Asan 51312, Asan 51843), type I-Rough (S-Mas_I-R; Asan 16 and Asan 22, Asan 34), type II-Rough (S-Mas_II-R; Asan 4 and Asan 50594, Asan 62188). All the strains were cultured from low-passage frozen stocks (at −70°C) to exponential phase and subcultured in 7H9 broth (supplemented with 10% ADC) or on 7H10 agar plates (supplemented with 10% OADC) at 37°C for each experiment. RGM were grown in Middlebrook 7H9 broth supplemented with Tween 80 and 10% ADC (BD) by shaking incubation for 3–5 days (SGM; for 14–28 days) at 37°C. To obtain single-cell bacterial suspensions, all the strains were washed and resuspended in phosphate-buffered saline (PBS) with 0.05% Tween 80 (PBS-T) and passed through a 27-gauge needle three to five times. The concentration was determined by measuring the optical density at 600 nm (OD 600) as a function of CFU/ml. To inactivate various mycobacteria strains, we heated them at 100°C in a boiling-water bath for 30 min.

### Confocal Microscopy Analysis

To evaluate co-localization with bacteria containing phagosome and lysosome, we prepared J774A.1 cells on chamber plates (Nunc™ Lab-Tek™ II Chamber Slide™ System). The cells infected for 24 h.p.i. with CFSE (#C34554, Invitrogen) stained bacteria [MAB-R (*M. abscessus* type strain ATCC 19977 rough strain) MAB-S (*M. abscessus* type strain ATCC 19977 smooth strain] at an M.O.I of 10. After infection, the cells were washed with PBS and then fixed with 4% paraformaldehyde for 10 min at room temperature. To visualize the phagosomal lysosome, the fixed infected cells were permeabilized with a 0.1% Triton X-100 for 10 min and blocked with 1% BSA for 30 min room temperature. The cells were labeled with rat anti-LAMP1 antibody (#25245, Abcam) and incubated for 18 h at 4°C and then labeled with Goat anti-Rat IgG (H+L), Alexa Fluor® 633 conjugate, dilution 1:1,000 for 45 min at room temperature. Nuclei were stained with 300 nM DAPI (#D1306, Invitrogen) for 5 min at room temperature. The samples were washed with PBS and mounted on a glass slide with Vectashield solution (Vector Laboratories) and observed using a confocal laser scanning microscope system (Olympus-FV1000).

### Construction of *Mycobacterium-E. coli* Shuttle Vectors for the Expression of EGFP

Using the pAL5000-TOPO vector, an EGFP-expressing *Mycobacterium-Escherichia coli* shuttle vector was generated as previous study (51). Briefly, the *EGFP* gene was amplified from the pIRES2-EGFP vector (Clontech, Mountain View, CA, USA; Cat No., 6029–1), and the *hsp65* promoter gene was amplified from genomic DNA of *M. bovis* BCG. The *EGFP* gene with the *hsp65* promoter was amplified from the pMyong2-EGFP^h^ vector and ligated into pAL5000-TOPO using *Hind*III and *Bam*HI restriction sites. The *hsp65*promoter and *EGFP* gene were amplified by overlapping PCR. To generate three different types of recombinant strains expressing EGFP (Asan 50594, *M. smegmatis* and BCG), the pAL5000-TOPO-EGFP plasmid was electroporated into competent bacteria (Asan 50594, *M. smegmatis* and BCG) using the Gene Pulser II electroporation apparatus (Bio-Rad, Hercules, CA, USA). Transformants were selected on Middlebrook 7H10 medium (Difco Laboratories, Detroit, MI, USA) (52) supplemented with OADC and containing 100 μg/ml kanamycin. Typically, colonies of transformants were selected from the plates, transferred into 7H9 broth medium (Difco Laboratories, Detroit, MI, USA) supplemented with 0.5% glycerol, 0.05% Tween-80, 10% ADC and 100 μg/ml kanamycin and cultured for 3~5 days. The growth rate of the recombinant mycobacteria strains was determined by measuring the medium OD at 600 nm.

### Infection With Mycobacterial Strains and Bacterial Counts

The J774A.1 cells (1.0 × 10^6^ cells), BMDMs (1.0 × 10^6^ cells), and immature BMDCs (1.0 × 10^6^ cells) were infected with mycobacterial strains at an M.O.I. of 10 (1 × 10^7^ bacteria) and incubated for 2 h to allow the phagocytosis of the bacteria. The infected cells were washed three times with PBS and to remove extracellular mycobacteria, cells were incubated with fresh culture medium containing 50 μg/ml amikacin (27, 53). The term h.p.i. (hours post infection) refers to the incubation time after a fresh antibiotic medium change. After further incubation, the cells were detached by PBS with 0.5% Triton-X-100 or by scraping at each time point. The cell pellets were diluted in PBS and plated onto 7H10 agar plates (supplemented with OADC) to determine the CFUs. The cell culture supernatants were collected and stored in a deep freezer (at −70°C) for determining the cytokine levels.

### Bacterial Staining

The bacterial infected cells were prepared on chamber plates (Nunc™ Lab-Tek™ II Chamber Slide™ System), and the plates were then placed on a dryer with their smeared surface upwards, where they were air-dried for approximately 30 min and then heat-fixed. The specimens were covered with carbol fuchsin stain and then heated until vapor just began to rise (i.e., approximately 60°C). Additional stain was added if necessary, and the heated stain remained on the slide for 5 min. The stain was removed by washing with clean water. The smear was covered with 3% v/v acid alcohol (or 20% sulfuric acid) for 20 s and washed off with clean water. Methylene blue was then applied to counterstain any cells that had been decolorized. The smear was then examined with microscope using the 100 × oil immersion objective (10 × eye piece for a total of 1,000 × magnification), and the smear was systematically scanned.

### Transmission Electron Microscopy

Infected cells were washed with PBS and fixed with 2% paraformaldehyde (v/v) and 2% glutaraldehyde (v/v) in 0.05 M Na cacodylate-HCl buffer (pH 7.2) at 4°C for 2~4 h. Primary fixed samples were washed three times with 0.05 M Na cacodylate-HCl buffer (pH 7.0) at 4°C for 10 min. The fixed samples were post fixed with 1% osmium tetroxide (w/v) in Na cacodylate-HCl buffer (pH 7.2) at 4°C for 2 h. Then, fixed samples were washed two times with distilled water at room temperature. The pelleted cells were block-stained with 0.5% uranyl acetate (w/v) at 4°C for 30 min and dehydrated with 30, 50, 70, 80, 90, 100, 100, and 100% at room temperature for 10 min each. The samples were transferred two times with 100% propylene oxide at room temperature for 15 min, 1:1 propylene oxide/Spurr's resin (2 h), 1:2 propylene oxide/Spurr's resin (4 h or overnight), and 100% Spurr's resin (2 h) to infiltrate. In the following step, samples were embedded in molds filled with a mixture of Spurr's epoxy resin and polymerized for 24 h at 70°C. The polymerized blocks were cut on a ultramicrotome (MT-X, RC, Tucson, AZ, USA), and sections were stained with 2% uranyl acetate (7 min) followed by Reynolds' lead citrate (7 min) and then examined on a JEOL transmission electron microscope (Japan) at 80 kV accelerating voltage.

### Immunoblotting Assay

Infected cell lysates were collected for protein analysis, and cells were lysed in RIPA buffer (Cell Signaling) containing complete EDTA-free protease inhibitor (Roche) and phosphatase inhibitor cocktail (Roche). Protein concentrations were determined using a bovine serum albumin (BSA) protein assay (Bio-Rad). Cellular proteins separated by SDS–polyacrylamide gel electrophoresis were electrotransferred to nitrocellulose (NC) membranes and subjected to immunoblot analysis with various primary antibodies. Immunoblot analysis was done with various primary antibodies: anti-rabbit IRF3 (#4302; Cell Signaling), anti-rabbit pIRF3 (#29047; Cell Signaling), anti-rabbit cGAS (#31659; Cell Signaling), anti-rabbit STING (#29047; Cell Signaling), anti-rabbit Actin (sc-1616; Santa Cruz Biotechnology), and anti-rabbit GAPDH (sc-25778; Santa Cruz Biotechnology) antibodies.

### Type I IFN Bioassay and Luciferase Reporter Assay

Supernatants from infected cells were overlaid on top of L929 IFN reporter cells containing the ISRE-luciferase construct (32) and incubated for 4 h (48- or 96-well plates). The reporter cells were lysed in 5 × Reporter Lysis Buffer (Promega, Madison, WI, USA) for 30 min at room temperature and mixed with firefly luciferin substrate (Promega, Madison, WI, USA), and luminescence was measured on an illuminometer (TECAN).

### Apoptosis Analysis

Cells were seeded in 12-well plates and infected the following day with the different strains as explained before. The supernatant was completely aspirated and washed with PBS 2 times, trypsinized cells were collected, and cells were used to analyze apoptosis by flow cytometry. Phosphatidylserine (PS) exposure and membrane integrity were analyzed by using Annexin V and 7-AAD (BD Biosciences) and flow cytometry according to the manufacturers' instructions. Briefly, cells were washed with PBS and incubated with Annexin V and 7-AAD in Annexin V Binding buffer for 15 min. Then, 400 μl of Annexin V Binding buffer was added to each tube, and the samples were analyzed by flow cytometry (FACS LSRFortessa X-20).

### Cellular Fractionation and Detection of Cytosolic Mycobacterial DNA

BMDM and J774A.1 cells were infected in 6-well plates. Cytosolic cell fractions were isolated using the manufacturer's protocol for the Qproteome Cell Compartment Kit (QIAGEN). Briefly, cell pellets were resuspended in ice-cold PBS by pipetting up and down and then centrifuged three times at 500 × *g* at 4°C for 10 min; this process was repeated three times. The supernatant was carefully removed and discarded, and the pellets were resuspended in Protease Inhibitor Solution lysis buffer, vortexed, and incubated at 4°C for 10 min on an end-over-end shaker. The lysate was centrifuged at 1,000 × *g* for 10 min at 4°C, and the supernatant was carefully transferred into a new microcentrifuge tube, where it was stored on ice. This supernatant primarily contained cytosolic proteins. To extract DNA from the fractionized supernatant, we performed the PCI method (54). To detect mycobacterial DNA, we specifically targeted the mycobacterial *hsp*65 gene (14) and applied PCR (55). A set of primers HspF3 (forward; 5′-ATC GCC AAG GAG ATC GAG CT-3′) and HspR4 (reverse; 5′-AAG GTG CCG CGG ATC TTG TT- 3′) was used.

### Enzyme-Linked Immunosorbent Assay

Cells were infected in 6-, 12-, and 24-well plates. Post infection cell culture supernatants were collected from infected cells and stored at −70°C. Paired antibodies and standard recombinant mouse IL-1β, TNF-α, IL-6, IL-10 (eBioscience), and IFN-β (BioLegend) were used to determine cytokine concentrations according to the manufacturers' instructions.

### RNA Purification and qRT-PCR

Total RNA was extracted from cells using TRIzol (Invitrogen) according to the manufacturer's instructions. For quantitative real-time PCR analysis, cDNA was amplified using specific primers for IFI204, IFNβ, STING, cGAS, IRF3, and SYBR green PCR Master Mix (Applied Biosystems, Grand Island, NY, USA) and processed using an ABI 7500 (Applied Biosystems, Grand Island, NY, USA). The results were acquired as cycle threshold (Ct) values and represented the average of at least two independent experiments. The relative amount of mRNA (2^ΔΔ*Ct*^) was obtained by normalizing its level to that of the β-actin gene in each experiment. Specific primers with the following sequences were used (33, 56): IFN-β forward 5′-ATGGTGGTCCGAGCAGAGAT-3′; IFN-β reverse 5′-CCACCACTCATTCTGAGGCA-3′; IFI204 forward 5′-GAGCAAGGCGGCTAAGGAA-3′; IFI204 reverse 5′-GCTGTGGAGTATTGGTGACTG-3′; STING forward 5′-AAATAACTGCCGCCTCATTG-3′; STING reverse 5′-ACAGTACGGAGGGAGGAGG-3′; IRF3 forward 5′-GGCTTGTGATGGTCAAGGTT-3′; IRF3 reverse 5′-CATGTCCTCCACCAAGTCCT-3′; β-actin forward 5′-GTGACGTTGACATCCGTAAAGA-3′; β-actin reverse 5′-GCCGGACTCATCGTACTCC-3′.

### Statistical Analysis

Statistical analysis was performed using GraphPad Prism 5 software and Microsoft Excel software. All experiments were performed at least two times and the data are presented as the mean ± standard deviation (SD). Asterisks indicate significance as determined by the ANOVA with Tukey's correction and the Student's *t*-test calculated as described in figure legends (^*^P < 0.05; ^**^P < 0.01, and ^***^P < 0.001).

## Ethics Statement

All animal treatment was in accordance with the institutional recommendations in the National Guidelines for the care and use of laboratory animals. The protocol was approved by the Institutional Animal Care and Use Committee (IACUC; approval No. of SNU-170717-7) of the Institute of Laboratory Animal Resources at Seoul National University.

## Author Contributions

B-RK and By-JK performed the experiments and analyzed the data. Bu-JK and Y-HK designed and interpreted the experiments. Bu-JK wrote the manuscript.

### Conflict of Interest Statement

The authors declare that the research was conducted in the absence of any commercial or financial relationships that could be construed as a potential conflict of interest.
