# Supplementary material for: Phagosome Escape of Rough Mycobacterium abscessus Strains in Murine Macrophage via Phagosomal Rupture Can Lead to Type I Interferon Production and Their Cell-To-Cell Spread
Source: Front Immunol. 2019 Jan 31;10:125. doi: 10.3389/fimmu.2019.00125 (PMC6365470; doi:10.3389/fimmu.2019.00125)
Supplement: Supplementary file 1 [file Data_Sheet_1.pdf]

Supplementary Figures

Phagosome escape of rough *Mycobacterium abscessus* strains in macrophage via phagosomal rupture can lead to Type I interferon production and their cell-to-cell spread

Bo-Ram Kim, Byoung-Jun Kim, Yoon-Hoh Kook and Bum-Joon Kim*

Department of Microbiology and Immunology, Biomedical Sciences, Liver Research Institute and Cancer Research Institute, College of Medicine, Seoul National University, Seoul, Korea

***Author for correspondence:** Bum-Joon Kim, phD,

E-mail : kbumjoon@snu.ac.kr.

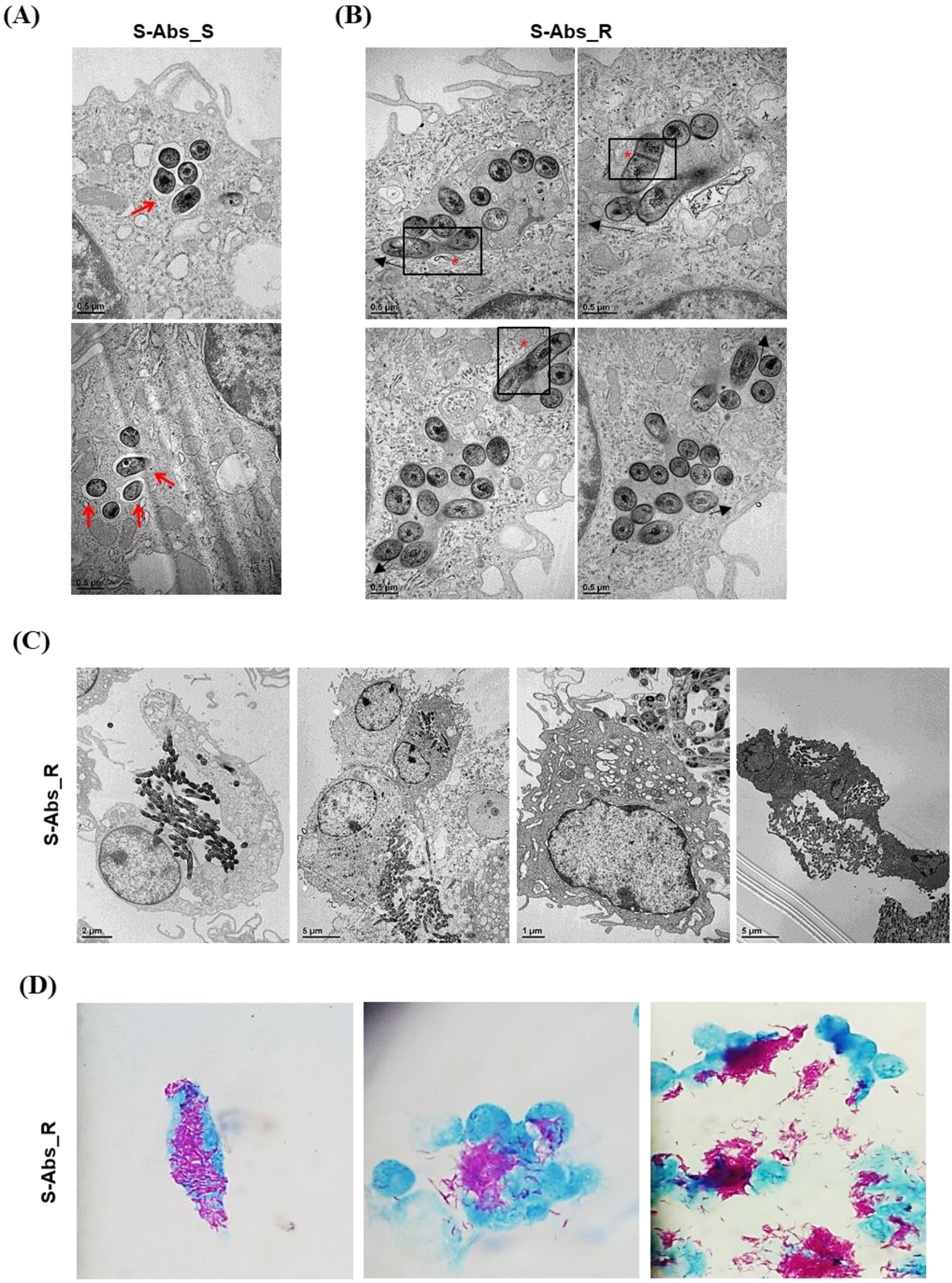
(A)
S-Abs_S
(B)
S-Abs_R
0.5 µm
(C)
S-Abs_R
2 µm
5 µm
1 µm
5 µm
(D)
S-Abs_R

(E)

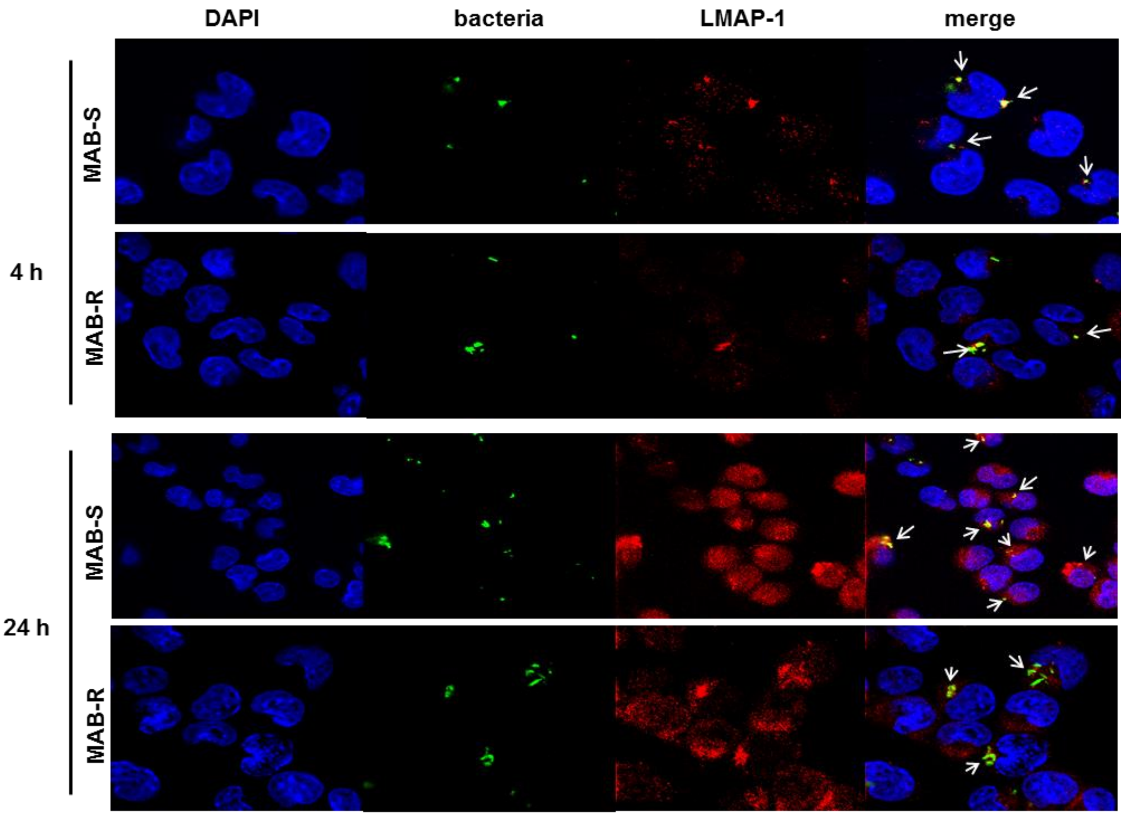

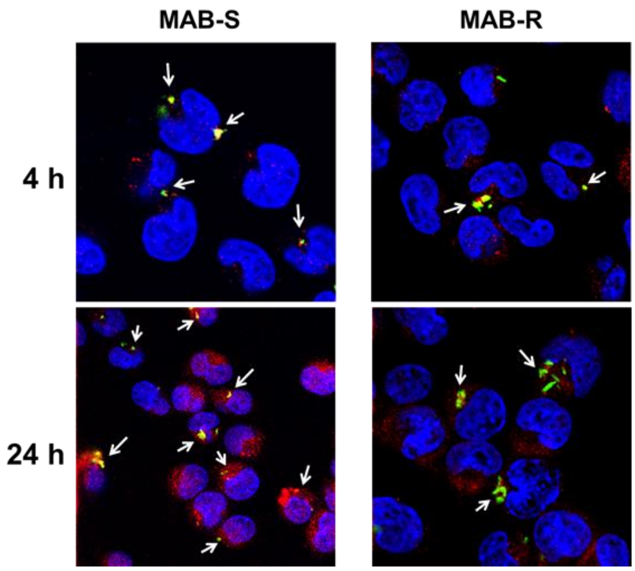

(F)

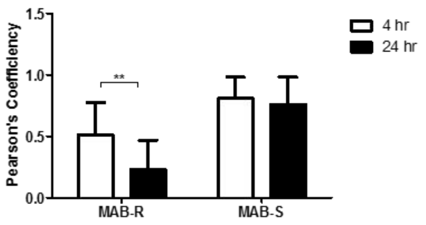

Figure S1. Comparison of bacterial escape into cytosol and co-localization with LAMP-1 between murine macrophages infected with MAB-R and MAB-S strains. (A and B) BMDM infected with S-Abs_S (*M. abscessus* type strain ATCC 19977 smooth strain) (A) and S-Abs_R (*M. abscessus* type strain ATCC 19977 rough strain) (B) (10 M.O.I.) for 24 h. TEM

analysis shows phagosome morphology images. We marked the bacterial replication status in the phagosome with a red asterisk. MAB-R strains divided into two bacteria in the phagosome (red asterisk) and were also removed from the phagosome to the (black arrow). Bar indicates 0.5 μm. (C) BMDM infected with S-Abs_R (*M. abscessus* type strain ATCC 19977 rough strain) (10 M.O.I.) for 24 h. TEM analysis shows the infected cell morphology and extracellular images of bacteria leaving. Bar indicates 1 or 2 or 5 μm. (D) AFB stain images of J774A.1 cells infected with S-Abs_R (*M. abscessus* type strain ATCC 19977 rough strain) (10 M.O.I.) for 24 h. All data are based on at least 20 cells per time point and are a representative result out of three independent experiments. (E) J774A.1 cells were infected with 10 M.O.I of CFSE (green) stained bacteria [MAB-R (*M. abscessus* type strain ATCC 19977 rough strain) MAB-S (*M. abscessus* type strain ATCC 19977 smooth strain] for 4 or 24 h.p.i. and then stained with DAPI (blue) and LAMP-1(red), representative images is shown. Non-infected J774A.1 cells were used as negative control. The white arrows showed co-localization of intracellular bacteria with LAMP-1 (yellow). (F) Quantification of Pearson's colocalization coefficient between MAB-R or -S and LAMP-1. The 20 bacteria randomly selected were analyzed and are a representative result out of two independent experiments. Results are means ± SD and $***P < 0.001$ (Student's t-test).

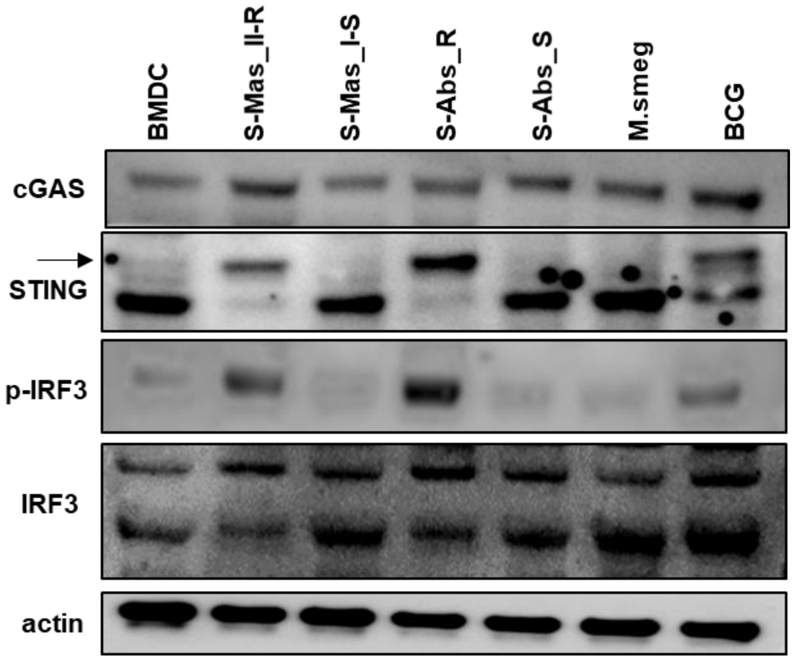

Figure S2. Western blot analysis of proteins related to Type I IFN induction in BMDCs. Rough strains [S-Abs_R (*M. abscessus* type strain ATCC 19977 rough strain) and S-Mas_II-R (Asan 50594)] and smooth strains [S-Abs_S (*M. abscessus* type strain ATCC 19977 smooth strain) and S-Mas_I-S (Asan 51843)], *M. smegmatis*, and BCG (10 M.O.I. infection) in BMDCs for 24 h. Preparation of total cell lysate for Western blot analysis. Briefly, the samples were run on 10% SDS-PAGE and treated with commercial anti-rabbit IRF3 (#4302; Cell Signaling), anti-rabbit pIRF3 (#29047; Cell Signaling), anti-rabbit cGAS (#31659; Cell Signaling), anti-rabbit STING (#29047; Cell Signaling), and anti-rabbit actin (sc-1616; Santa Cruz Biotechnology) antibodies.

(A)

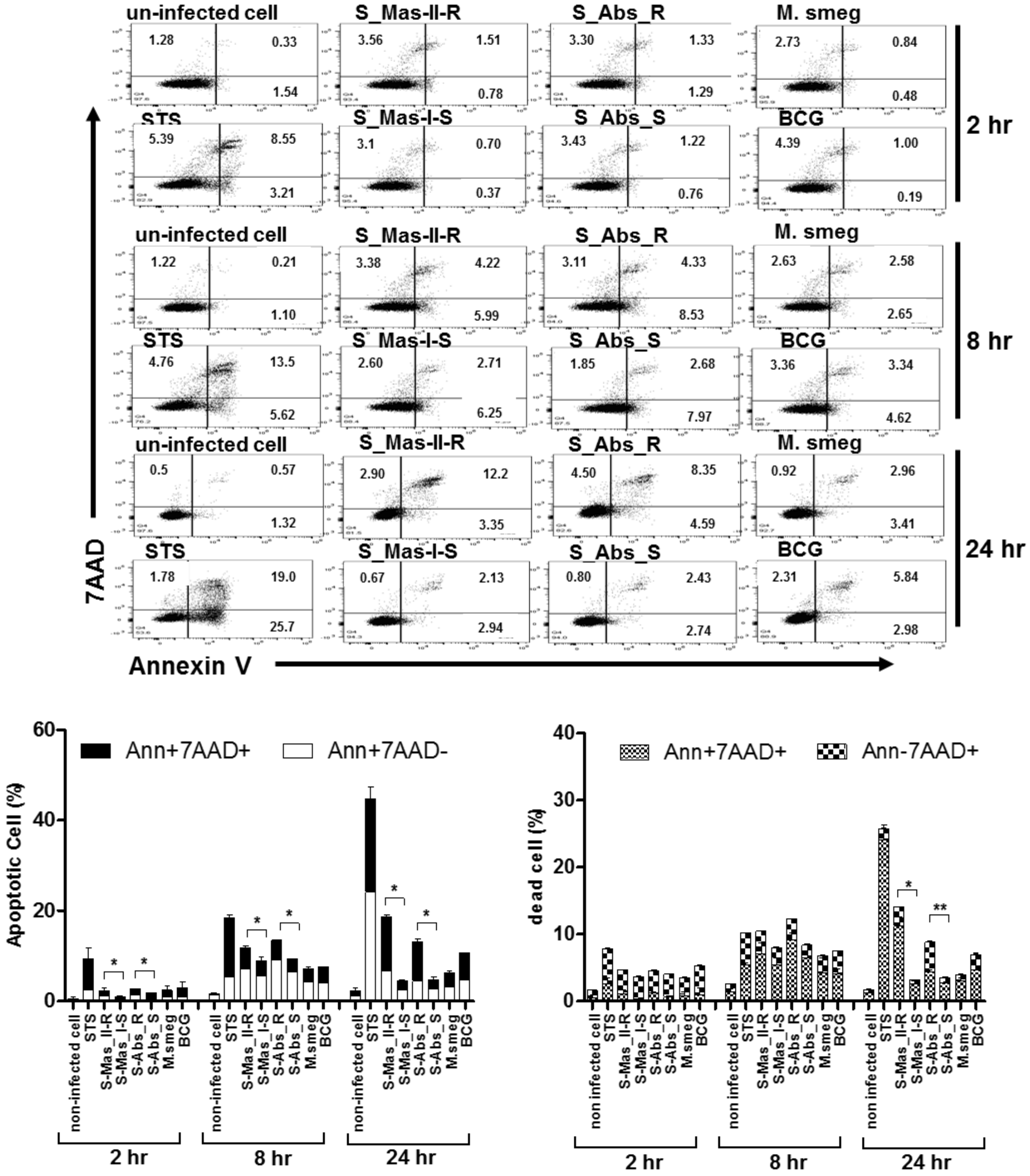
un-infected cell
S_Mas-II-R
S_Abs_R
M. smeg
STS
S_Mas-I-S
S_Abs_S
BCG
2 hr
8 hr
24 hr
1.28
0.33
1.54
3.56
1.51
0.78
3.30
1.33
1.29
2.73
0.84
0.48
5.39
8.55
3.21
3.1
0.70
0.37
3.43
1.22
0.76
4.39
1.00
0.19
1.22
0.21
1.10
3.38
4.22
5.99
3.11
4.33
8.53
2.63
2.58
2.65
4.76
13.5
5.62
2.60
2.71
6.25
1.85
2.68
7.97
3.36
3.34
4.62
0.5
0.57
1.32
2.90
12.2
3.35
4.50
8.35
4.59
0.92
2.96
3.41
1.78
19.0
25.7
0.67
2.13
2.94
0.80
2.43
2.74
2.31
5.84
2.98
7AAD
Annexin V
Ann+7AAD+
Ann+7AAD-
Apoptotic Cell (%)
Ann-7AAD+
dead cell (%)
non-infected cell
STS
S-Mas_II-R
S-Mas_I-S
S-Abs_R
S-Abs_S
M.smeg
BCG
2 hr
8 hr
24 hr

(B)

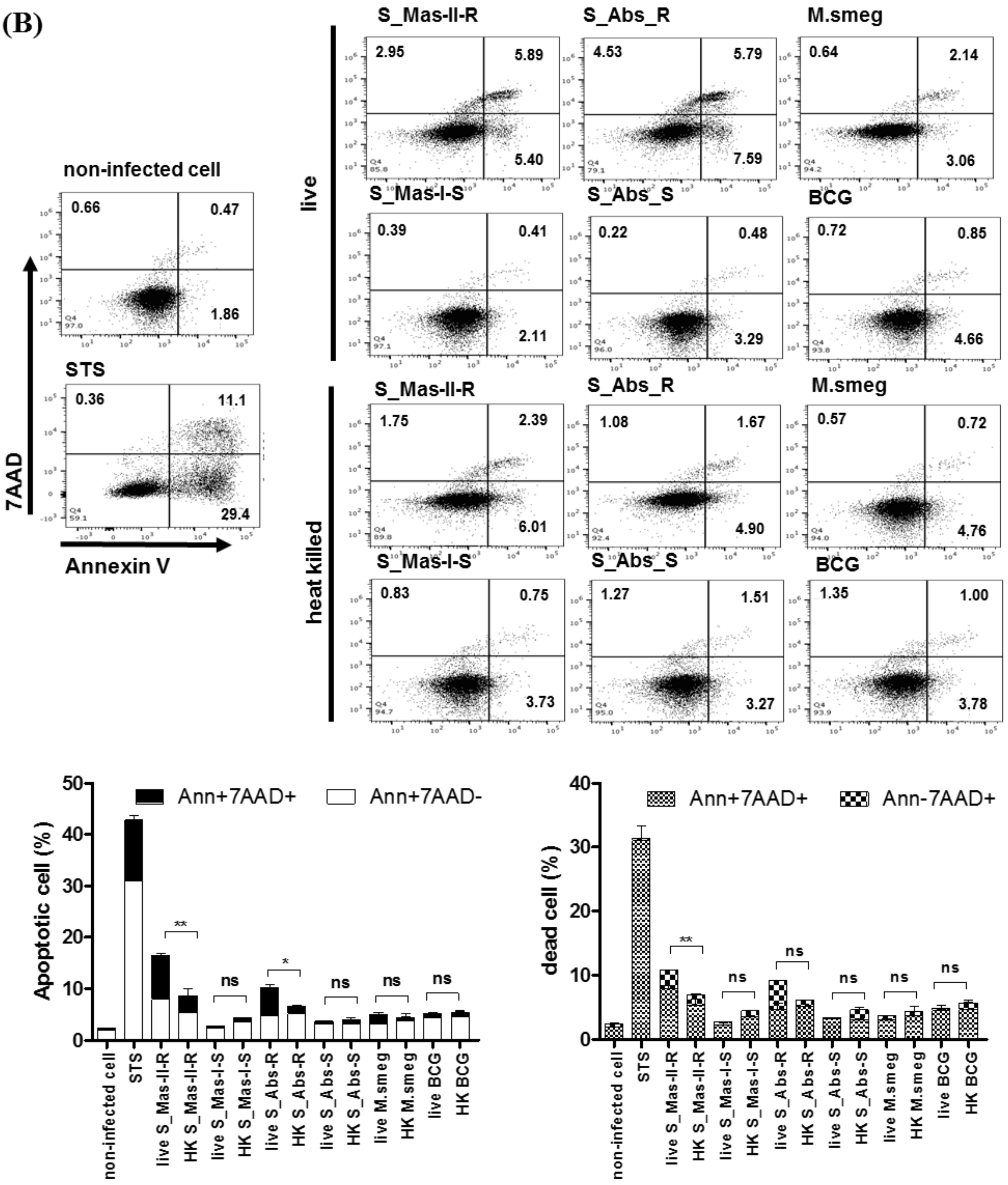

Figure S3. FACS analysis of Annexin V/7-AAD apoptosis assay on J774A.1 cell panel. (A) J774A.1 cells were pretreated with 100 nM staurosporine (apoptosis inducer) and infected with MAB-R strains [S-Abs_R (*M. abscessus* type strain ATCC 19977 rough strain) and S-Mas_II-R (Asan 50594)] and MAB-S strains [S-Abs_S (*M. abscessus* type strain ATCC 19977 smooth strain) and S-Mas_I-S (Asan 51843)], *M. smegmatis,* and *M. bovis* BCG (10 M.O.I) at different time points (2, 8 and 24 h). (B) J774A.1 cells were either uninfected (control) or infected with

live or heat-killed (HK) bacteria (10 M.O.I) of the indicated bacterial strains for 24 h. (A-B) The infected cells were stained with Annexin V and 7-AAD and analyzed by flow cytometry (FACS LSRFortessa X-20). The results are representative of two independent experiments and represent means ± SD. *P* values were determined by the Student's *t*-test using GraphPad prism program: ns, nonsignificant; *$P < 0.05$; **$P < 0.01$, and ***$P < 0.001$.

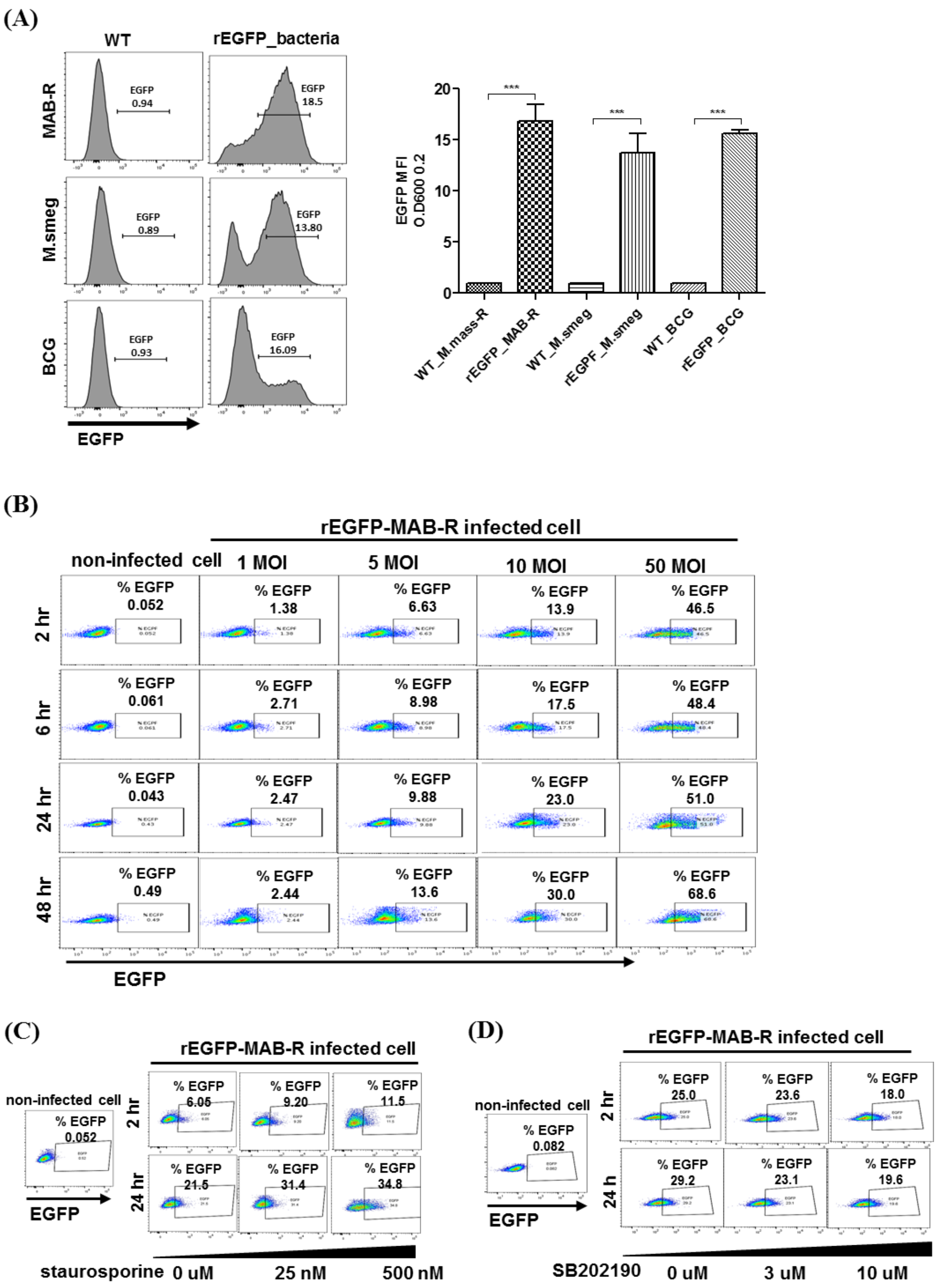

Figure S4. FACS analysis of recombinant mycobacteria expressing EGFP and cell-to-cell spread panel. (A) Recombinant mycobacteria expressing EGFP [rEGFP_MAB-R (Asan

50594), rEGFP_M.smeg, rEGFP_BCG) and wild-type mycobacteria were cultured for 3~5 days in 7H9 broth medium supplemented with 0.5% glycerol, 0.05% Tween-80, and 10% ADC, and 100 µg/ml kanamycin was added for recombinant mycobacteria. The growth rate of the recombinant mycobacterial strains was determined by measuring the OD value at 600 nm. Bacterial pellets were washed 3 times with PBS and resuspended in FACS buffer, and EGFP-positive cells were counted by FACScalibur (BD biosciences). (B) J774A.1 cells were infected with rEGFP_MAB-R and rEGFP M.smeg, analysis was performed on data from different time points (2, 6, 24, and 48 h) or M.O.I.s (1, 5, 10 and 50), and the percentage of EGFP-positive cells was measured by flow cytometry (FACScalibur). (C-D) J774A.1 cells were pretreated with staurosporine (apoptosis inducer) or SB202190 (p38 MAPK inhibitor) and infected with MAB-R [rEGFP_MAB-R (Asan 50594)] (10 M.O.I.), and the percentage of EGFP-positive cells was measured by flow cytometry (FACScalibur). The results are representative of two independent experiments and represent means ± SD. *P* values were determined by the Student's *t*-test using GraphPad prism program: ns, nonsignificant; $*P < 0.05$; $**P < 0.01$, and $***P < 0.001$.

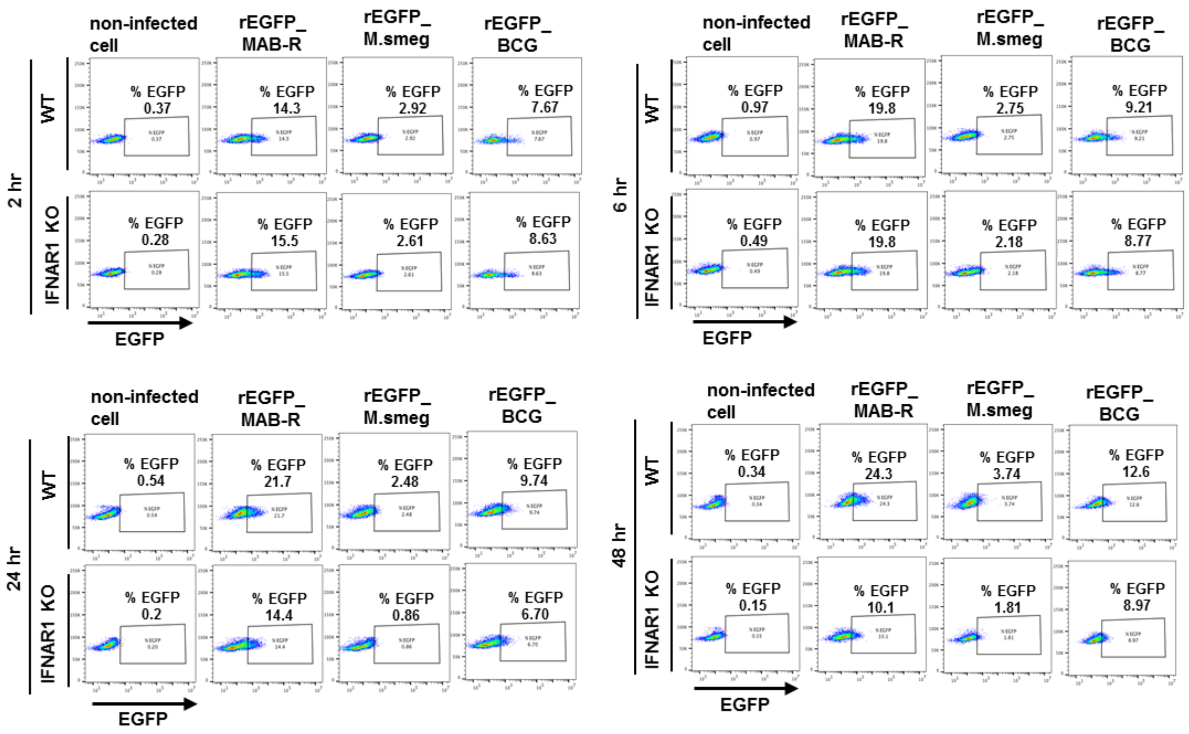

Figure S5. FACS analysis of the cell-to-cell spread during MAB-R infection depends on Type I IFN signaling. BMDMs (WT and IFNAR1 KO mice) infected with EGFP-expressing bacteria [rEGFP_MAB-R (Asan 50594), rEGFP_M.smeg, rEGFP_BCG] (10 M.O.I) for different times (2, 6, 24 and 48 h). The supernatant was completely aspirated and washed with PBS 3 times, and trypsinized cells were collected. Cells and the percentage of EGFP-positive cells were analyzed by flow cytometry (FACScalibur).

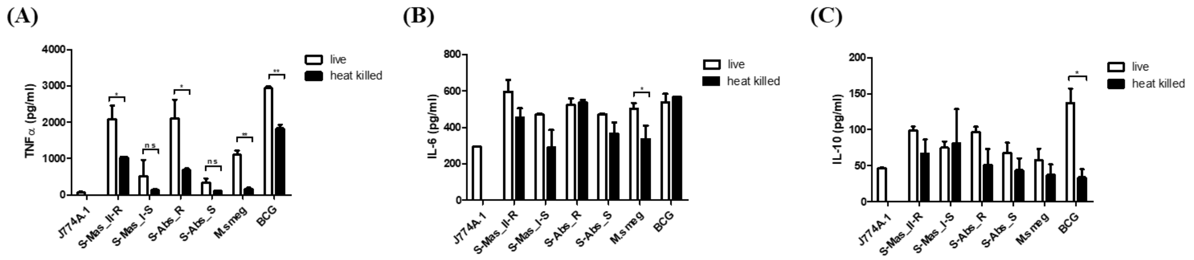

Figure S6. Cytokine production by live or heat-killed mycobacteria in murine macrophages. Live or heat-killed MAB-R strains [S-Abs_R (*M. abscessus* type strain ATCC 19977 rough strain) and S-Mas_II-R (Asan 50594)] and MAB-S strains [S-Abs_S (*M. abscessus* type strain ATCC 19977 smooth strain) and S-Mas_I-S (Asan 51843)], *M. smegmatis*, and BCG (10 M.O.I. infection) infecting J774A.1 cells for 24 h. Infected cell supernatants and TNF-α IL-6 andIL-10 cytokine levels were analyzed by ELISA. The results are representative of two independent experiments and represent means ± SD. *P* values were determined by the Student's *t*-test using GraphPad prism program: ns, nonsignificant; *$P < 0.05$; **$P < 0.01$, and ***$P < 0.001$.
